# Inflammatory bowel disease and risk of autoimmune hepatitis: A univariable and multivariable Mendelian randomization study

**DOI:** 10.1371/journal.pone.0305220

**Published:** 2024-06-07

**Authors:** Gang Chi, Jinhong Pei, Xueqing Li

**Affiliations:** Department of Biochemistry, Changzhi Medical College, Changazhi, Shanxi, China; University Hospital of Bologna Sant’Orsola-Malpighi Polyclinic Department of Digestive System: Azienda Ospedaliero-Universitaria di Bologna Policlinico Sant’Orsola-Malpighi Dipartimento dell’apparato digerente, ITALY

## Abstract

**Objective:**

This study aimed to use Mendelian randomization (MR) to investigate the potential causal association between inflammatory bowel disease (IBD) and autoimmune hepatitis (AIH).

**Methods:**

Two-sample MR was performed to estimate the causal effect of IBD on AIH. The primary analysis employed the inverse variance weighted (IVW) method in univariable MR analysis, supplemented by additional methods including MR-Egger, weighted median, simple mode, and weighted mode. The *p* values were adjusted by FDR *p*-value adjustment. In the replication analysis, the primary IVW analysis was repeated and then pooled by meta-analysis. Sensitivity analyses were performed using Cochran’s Q test, MR-Egger intercept test, MR-PRESSO, leave-one-out, and funnel plot analysis to evaluate the robustness of the MR findings. Additionally, multivariable MR (MVMR) was employed to estimate the direct causal effect of IBD on the risk of AIH.

**Results:**

In univariable MR analysis, a significant positive causal association was observed between IBD (both Crohn’s disease (CD) or ulcerative colitis (UC)) and the risk of AIH (for CD and AIH, the IVW odds ratio (OR) = 1.10, 95% confidence interval (CI) = 1.00–1.16, *P* = 0.045, *FDR P* = 0.045; for UC and AIH, the IVW OR = 1.07, 95% CI = 1.00–1.13, *P* = 0.038, *FDR P* = 0.076). Furthermore, no significant positive correlation between IBD and the risk of AIH (OR = 1.13, 95% CI = 0.94–1.35, *P* = 0.194). Sensitivity analysis revealed no pleiotropic bias. MVMR analysis further confirmed the direct causal effect of CD or UC on the risk of AIH after adjusting for the common risk factors (cigarettes per day and osteoporosis). In the replication analysis, the positive causal association between UC and the risk of AIH remain significant (the IVW odds ratio (OR) = 1.32, 95% CI = 1.18–1.48, *P* = 2.90E-06). While no significant positive association was observed between CD or IBD and the risk of AIH in the replication analysis, a suggestive positive association between the identified risk factors (UC, CD, and IBD) and the risk of AIH was detected in the meta-analysis (OR = 1.09, 95% CI = 1.05–1.13, P<0.0001).

**Conclusion:**

This MR study revealed a positive impact of the identified risk factors (CD, UC and IBD) on the risk of AIH within the European population.

## Introduction

Autoimmune hepatitis (AIH) is a chronic progressive inflammatory liver disease mediated by the immune system, which is characterized by circulating autoantibodies, hyperglobulinemia, and histological interface hepatitis [1]. Its global incidence and prevalence are reported as 1.37 and 17.44 per 100,000 people every year, respectively, with a rising trend in incidence [2]. AIH is recognized as a disease that impacts individuals of all genders, ages, and ethnicities worldwide, although it predominantly affects females [3]. The ultimate clinical and histological characteristics of the disease remain consistent regardless of the age of onset. However, older patients typically exhibit distinct serological and genetic features and often present as asymptomatic or with insidious symptoms, frequently accompanied by autoimmune hypothyroidism. In older patients, the prevalence of HLA-DR4 and antinuclear antibody positivity is significantly higher compared to younger patients. Furthermore, more than a quarter of older patients (aged ≥65) are diagnosed with AIH [4, 5]. Consequently, early detection of AIH is important, especially in the elderly population, as immunosuppressive treatment may prove to be more effective and better tolerated.

AIH exhibits a global prevalence, with varying genetic predispositions observed among different regions and ethnicities. In patients with type 1 AIH from North America and Northern Europe, genetic susceptibility is associated with HLA A1-B8-DR3 and HLA DR4. Notably, the B8-DR3-DQ2 phenotype is prevalent among Italian patients with AIH-1, whereas HLA DR4 assumes a secondary role in North American and Northern European populations. DRB1*0404 emerges as the primary susceptibility allele for Mexican Mestizo patients. Conversely, DRB1*0405 exhibits a strong association with Argentina and Japanese adults. In the Brazilian population, the major HLA allele associated with AIH is DRB1*1301 [6, 7]. Therefore, genetic susceptibility potentially influences disease progression and treatment outcomes.

AIH presents as a non-self-limiting condition that, if left untreated, can progress to cirrhosis and fulminant hepatic failure, posing a serious threat to human health [8]. The primary treatment for AIH involves non-specific immunosuppression, leading to remission in a majority of patients. However, long-term immunosuppressive therapy is linked to a broad spectrum of adverse effects and prolonged morbidity [9]. Furthermore, a significant proportion of patients exhibit an inadequate response to immunosuppressive treatment, persisting with an inflammation response [10]. Liver transplantation is the most effective recourse for non-responder patients with AIH advancing to liver failure. Unfortunately, reports indicate that AIH recurs in 10% of patients within the initial year post-liver transplant and in 36–68% within 5 years thereafter [11]. The absence of specific and targeted treatments, alongside reliance on non-specific immunosuppressive agents for AIH, may contribute to a poor prognosis. Hence, there is a pressing need to investigate novel ideas and methodologies for the prevention and treatment of AIH.

Inflammatory bowel disease (IBD) is a chronic inflammatory condition affecting the intestines, primarily categorized as Crohn’s disease (CD) and ulcerative colitis (UC). Up to 50% of patients with IBD experience extra-intestinal manifestations, with hepatobiliary disorders ranking among the most prevalent [12]. Numerous case-control studies confirmed the presence of UC in 16% of patients with AIH [13, 14]. Observational studies have further indicated that the incidence of IBD reaches as high as 40–50% in patients diagnosed with primary sclerosing cholangitis (PSC)-AIH overlap syndrome, whereas PSC-AIH overlap syndrome remains rare among patients with IBD [15–17]. Identifying and treating IBD associated with AIH could potentially aid in the prevention or treatment of AIH. However, the potential causal relationship between IBD and AIH remains undetermined.

Conventional observational studies were susceptible to residual confounding and reverse causality bias [18]. Mendelian randomization (MR) employed single nucleotide polymorphisms (SNPs) as instrumental variables (IVs) of exposure to evaluate the causal effect of exposure on the outcome. Consequently, MR analysis was less susceptible to conventional confounders and reduced bias in observational studies [19]. Generally, the gold standard for evaluating the causal effect of exposure on an outcome is the randomized controlled trial (RCT). However, conducting RCTs can be challenging and expensive due to the complex experimental designs, cumbersome and time-consuming processes, and strict ethical requirements [20]. MR is regarded as a natural RCT because the random allocation of alleles at meiosis parallels the RCT’s design [21]. Therefore, MR serves as a valuable complement to RCTs by utilizing genetic data as instruments to make causal inferences, offering insights into establishing causal relationships in situations where conducting RCT studies is difficult.

This study aimed to employ a two-sample MR approach to investigate the potential causal association between genetic predisposition for CD and UC and the risk of AIH.

## Materials and methods

### Study design

**Fig 1:**
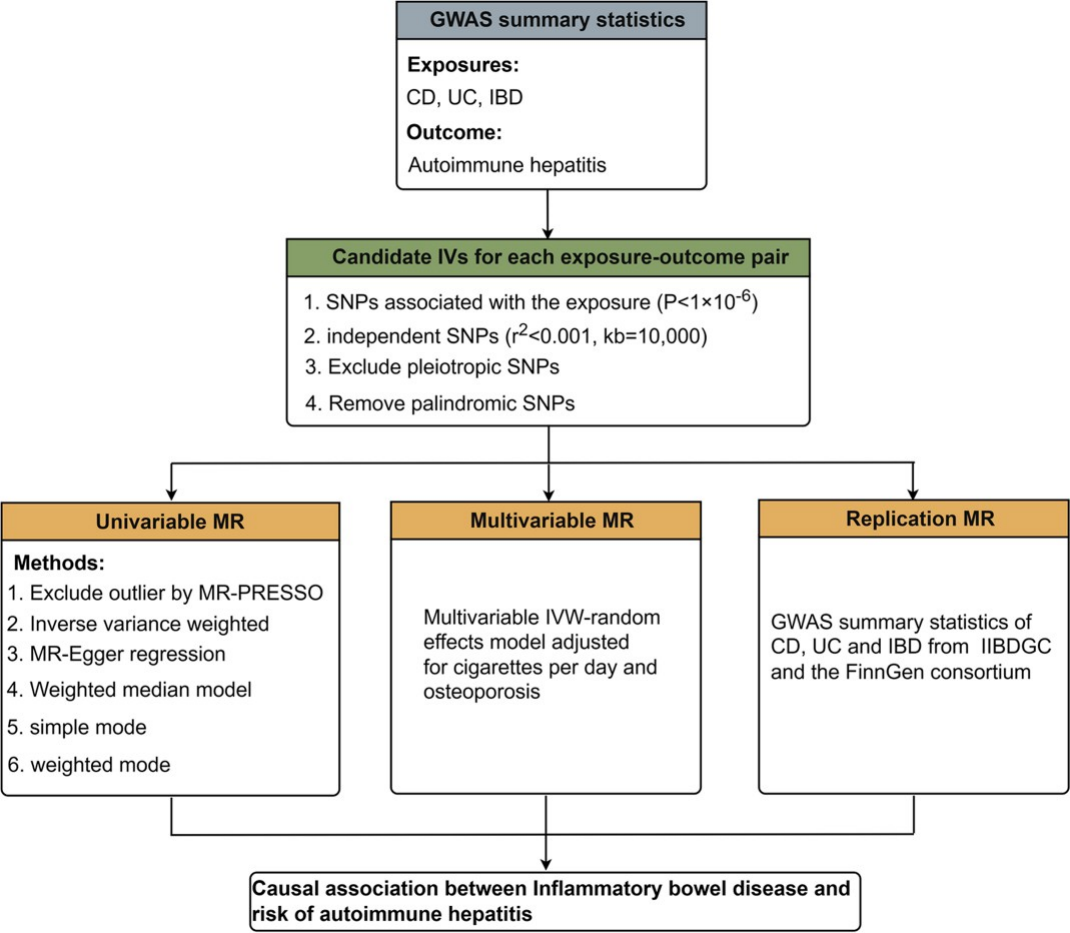
The study design overview and flowchart of MR analysis in this study. GWAS, genome-wide association studies; SNP, single nucleotide polymorphisms; IVs, instrument variables; MR, Mendelian randomization; PRESSO, pleiotropy residual sum and outlier; IVW, inverse variance weighted; CD, Crohn’s diseaseor; UC, ulcerative colitis; IBD, inflammatory bowel disease.

The overview of this study design was presented in Fig 1. First, we obtained the summary data of exposures (CD, UC and IBD) and outcome (AIH) from the genome-wide association study (GWAS). Second, the univariable two-sample MR was performed to estimate the causal effect between exposures and outcome. Third, the multivariable MR (MVMR) analysis was conducted adjusting for cigarettes per day and osteoporosis. Finally, we performed the replication analysis to further validate the initial MR results.

## Data source

Summary-level genetic associations with UC and AIH were extracted from the genome-wide association studies (GWAS) catalog. Specifically, for UC (GCST000964), data included 6,687 cases and 19,718 controls of European ancestry [22]. Additionally, information on 1,690 CD cases and 3,719 controls of European ancestry were derived from the International IBD Genetics Consortium (IIBDGC) [23]. GWAS summary data for IBD sourced from the IIBDGC with 5,673 cases and 213,119 controls [23]. The complete GWAS summary statistics for cigarettes per day were obtained from the GWAS and Sequencing Consortium of Alcohol and Nicotine use encompassing data from 337,334 individuals of European ancestry [24]. Additionally, GWAS data for osteoporosis were retrieved from the Integrative Epidemiology Unit (IEU) Open GWAS database (ID: finn-b-M13_OSTEOPOROSIS), consisting of 3,203 cases and 209,575 controls of European ancestry [25]. For AIH (GCST90018785), the dataset comprised 821 cases and 484,413 controls of European ancestry, as reported in GWAS results [26]. Specifically, a strong signal associated with AIH was fine-mapped to HLA-DRB1 Lys71 using data from the UK Biobank. However, diagnostic biomarkers specifically linked to HLA-DRB1 Lys71 were not reported. Nonetheless, several biomarkers associated with HLA-DRB1 have been reported, including diastolic blood pressure, triglycerides, glycated hemoglobin, total protein, albumin, blood urea nitrogen, calcium, aspartate transaminase, alanine aminotransferase, alkaline phosphatase, γ-glutamyl transpeptidase, C-reactive protein, white blood cell, neutrophil, eosinophil, monocyte, and lymphocyte [26].

Considering the stability of the results in MR analysis, we replicated the primary analysis using the aforementioned UC and CD GWAS data and utilized the latest publicly available data for CD from IEU Open GWAS (finn-b-CHRONLARGE), consisting of 807 cases and 210,300 controls of European ancestry. Similarly, we employed recent GWAS summary data for UC from the FinnGen consortium R10, comprising 246 cases and 398,152 controls of European ancestry. Additionally, we incorporated GWAS summary data for IBD into the replication analysis, sourced from the IIBDGC with 25,042 cases and 34,915 controls [23]. Subsequently, we performed a meta-analysis of the primary and replication samples to consolidate our findings. Detailed information regarding the genetic instruments utilized in the analysis is provided in S1 Table.

### Genetic instrument selection

SNPs that demonstrated significant associations with *P*<1×10^−6^ were identified. Independent SNPs were further evaluated by clustering SNPs in linkage disequilibrium (r2>0.001 or within 10,000 kb). Subsequently, selected SNPs were harmonized, with ambiguous and palindromic SNPs excluded from analysis. F statistic >10 was used to confirm the strength of the IVs to overcome weak instrument bias [27]. These SNPs were designated as genetic instruments utilized to adjust for the effects of the common risk factors (cigarettes per day and osteoporosis) through multivariable MR (MVMR) analysis.

### Statistical analysis

The inverse variance weighted (IVW) method was used as the primary analysis method, complemented by other MR methods, including MR-Egger, weighted median, simple mode, and weighted mode. The IVW method operated under the assumption that each SNP could be treated as a valid IV, combining the Wald ratio estimates of each SNP to generate a cumulative causal estimate. Acknowledging the challenge of verifying the validity of this assumption, MR-Egger and weighted median methods were implemented as supplementary analyses. These methods offer more robust estimates across a diverse array of scenarios, enhancing the reliability of the findings [28]. The MR-Egger method was used to assess the presence of horizontal pleiotropy among selected IVs. MR-Egger employed weighted linear regression of the gene-outcome coefficients on the gene-exposure coefficients. In this regression, the slope represented the estimate of the causal effect, while the intercept provided an estimate of the average horizontal pleiotropic effect across the genetic variants [29, 30]. The weighted median method yielded a consistent estimate of the causal effect when over 50% of the information was derived from valid IVs. Comparatively, the weighted median estimator provided more precise estimates than the MR-Egger analysis [31]. Additionally, the weighted mode proved effective in scenarios where a majority of instrumental variables were valid, even in the presence of other instrumental variables that might not fulfill the prerequisites of MR causal inference [32]. The simple mode served as an assessment approach designed to enhance robustness against pleiotropy [33]. Moreover, the MR-PRESSO method was employed to address potential pleiotropic outliers within the generated MR model. Outliers were identified and removed, followed by re-estimation of the casual effect [34]. The analysis was considered statistically significant at *P*<0.05. The consistency of effects observed across different methods reinforced the evidence for causality, as each method operates under its own set of assumptions. We implemented MVMR analysis, adjusting for cigarettes per day and osteoporosis, to determine the independent effects of IBDs on AIH. The significance level of *P*<0.05 was utilized for statistical inference. The *p* values were adjusted by false discovery rate (FDR) *p*-value adjustment. All analyses were performed in the R program (version 4.2.1) using the “TwoSampleMR” package (version 0.5.5), the “Mendelian Randomization” package (version 0.5.1), “MRPRESSO” package (version 1.0), and the “MVMR” package (version 0.3).

### Sensitivity analysis

The MR-Egger intercept test was used to assess horizontal pleiotropy to verify whether a single locus influenced multiple phenotypes. The results were visualized through scatter plots. Additionally, Cochran’s Q statistics were used to examine heterogeneity, with the outcome depicted using funnel plots. To identify and address the horizontal pleiotropic outliers and resolve detected heterogeneity, MR-PRESSO was applied. Sensitivity analysis was performed based on the leave-one-out approach.

## Results

### Genetic association between CD and AIH

**Fig 2:**
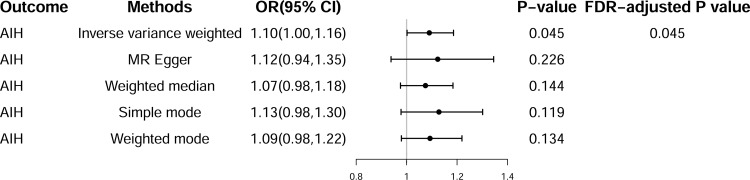
Summary of univariate Mendelian randomization (MR) analyses of the causal effects between Crohn’s disease (CD) and the risk of autoimmune hepatitis (AIH). OR, odds ratio; CI, confidence interval; FDR, false discovery rate.

**Fig 3:**
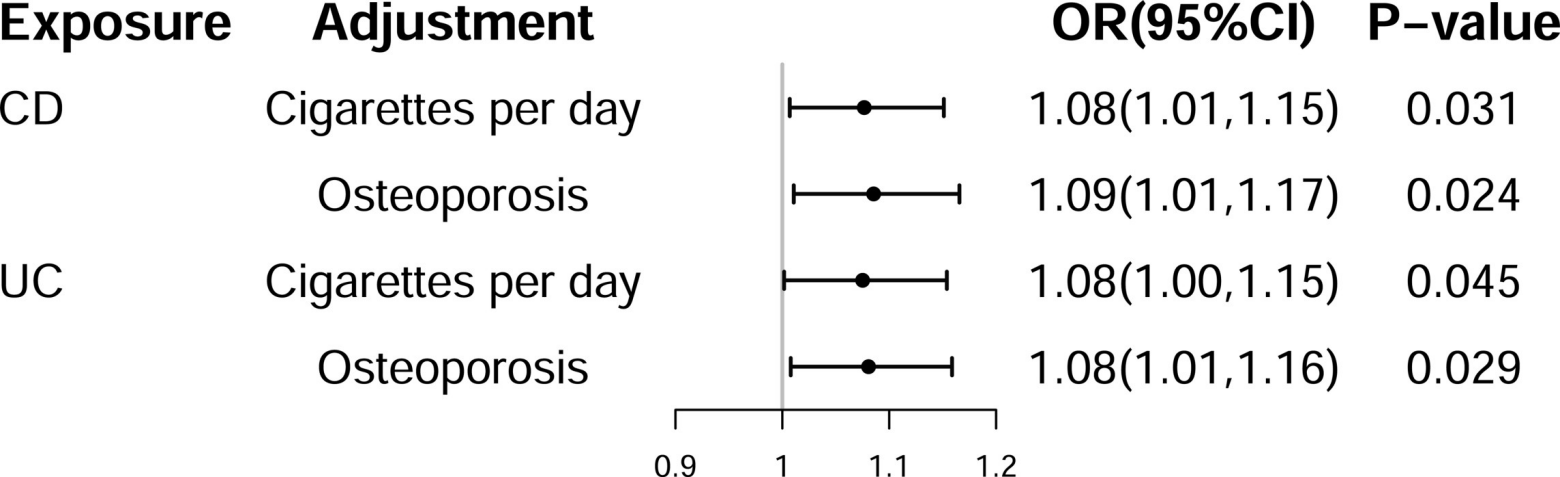
Summary of multivariable Mendelian randomization (MR) analyses of the causal effects between Crohn’s disease (CD) or ulcerative colitis (UC) and the risk of autoimmune hepatitis (AIH) using inverse variance weighted (IVW) method. OR, odds ratio; CI, confidence interval.

Sixteen SNPs were utilized to represent the genetic effects of CD (S1 Table). The IVW method indicated a positive causal association between CD and the risk of AIH (odds ratio (OR) = 1.10, 95% confidence interval (95% CI) = 1.00–1.16, *P* = 0.045), and the association remained statistically significant following false discovery rate (FDR) correction (FDR-adjusted *P* = 0.045) (Figs 2 and S1). Furthermore, the genetic association was not significant in other analyses, including MR-Egger, weighted median, simple mode, and weighted mode, although they consistently indicated the same directionality with IVW (Fig 2). Heterogeneity analyses revealed no significant heterogeneity among the selected IVs evaluated by IVW (Cochran’s Q = 23.24; *P* = 0.08) and MR-Egger (Cochran’s Q = 23.01; *P* = 0.06). Additionally, the MR-Egger pleiotropy test indicated the absence of pleiotropic bias in evaluating the effect of CD on the risk of AIH using the IVW method. Although the funnel plot exhibited some asymmetry, the Cochran’s Q test did not indicate significant heterogeneity. This suggested that the observed funnel plot asymmetry may not be indicative of substantial pleiotropy. Additionally, we employed the MR-Egger intercept test to further assess the presence of horizontal pleiotropy (Table 1 and S1 Fig). Besides, the MR-PRESSO analysis revealed no outlier SNPs (Table 1). Leave-one-out analysis demonstrated that the relationship between CD and the risk of AIH was not driven by any individual SNP (S1 Fig). Previous studies have highlighted smoking and osteoporosis as risk factors for both IBD and AIH [35–37]. Therefore, MVMR analysis was conducted to adjust for the effects of smoking and osteoporosis and to assess for potential confounding. The MVMR analysis indicated the direct effect of genetic liability to CD on the risk of AIH (Fig 3).

**Table 1:** Sensitivity analysis of the MR analysis result of exposure and outcome.

Exposure	Outcome	Heterogeneity test		Pleiotropy test	MR-PRESSO	
		Cochran’s Q (P value)	Rucker’s Q (P value)	Egger intercept (P value)	Distortion test	Global test
		IVW	MR Egger	MR Egger	Outliers	P value
CD	AIH	0.08	0.06	0.71	NA	0.06
UC	AIH	0.32	0.30	0.67	NA	0.31
IBD	AIH	0.14	0.17	0.22	NA	0.17

### Genetic association between UC and AIH

**Fig 4:**
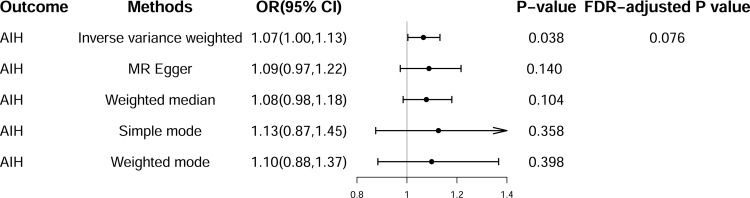
Summary of univariate Mendelian randomization (MR) analyses of the causal effects between ulcerative colitis (UC) and the risk of autoimmune hepatitis (AIH). OR, odds ratio; CI, confidence interval; FDR, false discovery rate.

A total of 131 SNPs were employed as IVs to evaluate the effect of UC (S1 Table). The IVW method indicated a significant positive correlation between UC and the risk of AIH (OR = 1.07, 95% CI = 1.00–1.13, *P* = 0.038). However, this correlation was not significant following FDR correction (FDR-adjusted *P* = 0.076), likely owing to the limited sample size (Figs 4 and S1). Furthermore, MR-Egger, weighted median, simple mode, and weighted mode analyses did not reveal a significant positive association between UC and the risk of AIH. Nonetheless, the direction of the results remained consistent with the primary IVW method (Fig 4). MVMR analysis indicated that UC was an independent risk factor for the risk of AIH after adjusting for smoking and osteoporosis (Fig 3). The sensitivity analysis revealed no heterogeneity, pleiotropy, or outliers (Table 1). Although the funnel plot was not completely symmetrical, the Cochran’s Q test did not reveal significant heterogeneity, suggesting that the funnel plot asymmetry was not indicative of substantial pleiotropy. Moreover, MR-Egger intercept test was used to further evaluate the potential for horizontal pleiotropy (Table 1 and S1 Fig).

### Genetic association between IBD and AIH

**Fig 5:**
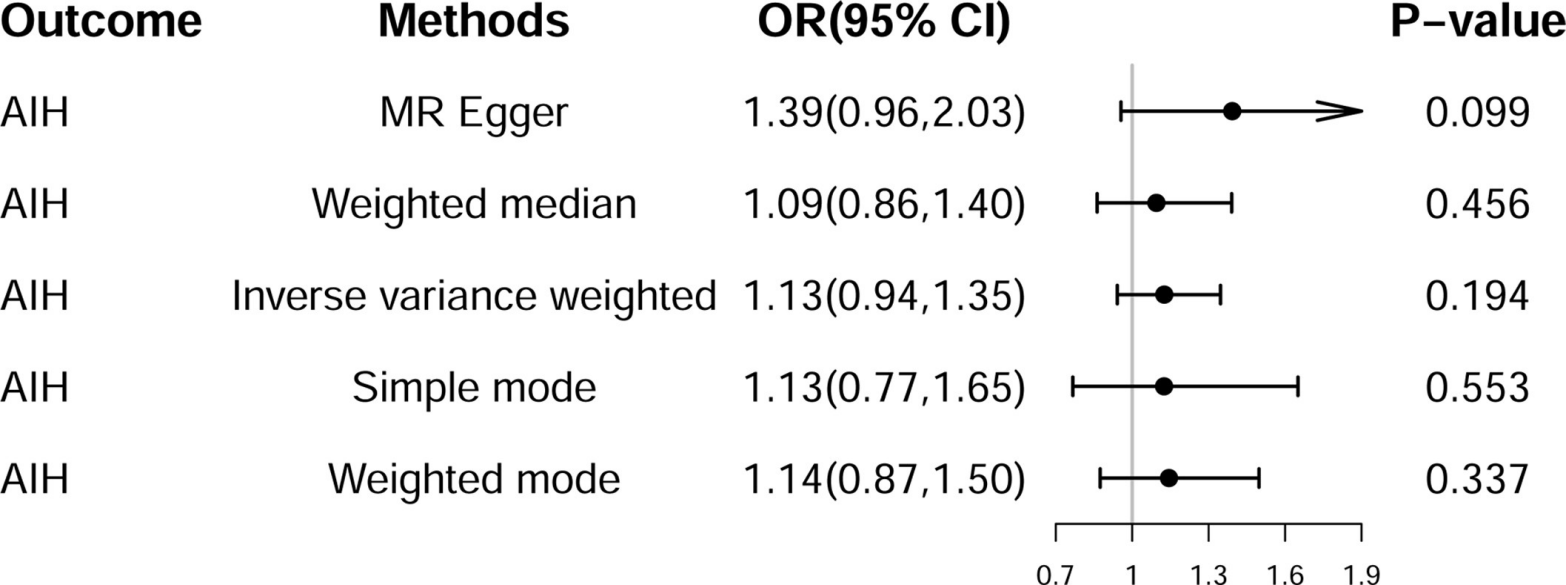
Summary of univariate Mendelian randomization (MR) analyses of the causal effects between inflammatory bowel disease (IBD) and the risk of autoimmune hepatitis (AIH). OR, odds ratio; CI, confidence interval.

A total of 23 SNPs were employed as IVs to evaluate the genetic effect of IBD on the risk of AIH (S1 Table). The IVW method indicated no significant positive correlation between IBD and the risk of AIH (OR = 1.13, 95% CI = 0.94–1.35, *P* = 0.194) (Figs 5 and S1). Moreover, other analyses were also not significant positive association, including MR-Egger, weighted median, simple mode, and weighted mode. However, the direction of the effect was consistent across all five methods (Fig 5). The sensitivity analysis revealed no heterogeneity, pleiotropy, or outliers (Table 1). The funnel plot was roughly symmetrical (S1 Fig).

## Results of the replication

**Fig 6:**
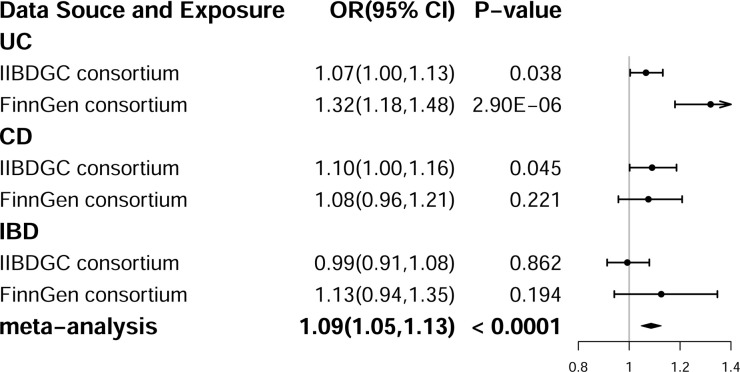
The effect of risk factors (Crohn’s disease (CD), ulcerative colitis (UC), and inflammatory bowel disease (IBD)) on the risk of autoimmune hepatitis (AIH) in replication analysis and meta-analysis. OR, odds ratio; CI, confidence interval.

To investigate the consistency of the association between IBD and the risk of AIH across diverse data sources, the primary IVW analysis was repeated using genetic associations from both the IIBDGC and the FinnGen consortium. The positive effect of UC on the risk of AIH was effectively replicated, with the direction of the effect remaining consistent with that observed in the discovery stage (Figs 6 and S1). In the replication analysis conducted using data from the FinnGen consortium, no association was observed between CD and the risk of AIH compared to the findings from the discovery stage involving the IIBDGC; however, the direction of the effect remained consistent with that observed in the discovery stage (Figs 6 and S1). Conversely, the association between IBD and the risk of AIH was not replicated in either the IIBDGC or the FinnGen consortium datasets, and notably, the direction of the effect differed between the two consortia (Figs 6 and S1). Furthermore, a suggestive positive association between the identified risk factors (UC, CD, and IBD) and an increase in the risk of AIH was detected in the meta-analysis (OR = 1.09, 95% CI = 1.05–1.13, *P*<0.0001), which further supported the results in the discovery stage (Fig 6).

## Discussion

In this study, GWAS data was used to investigate the potential causal relationship between CD or UC and the risk of AIH. Our findings indicate that CD and UC exert a positive causal effect, contributing to an increase in the risk of AIH, as revealed through two-sample MR analysis. Moreover, the significant effect of CD or UC on the risk of AIH persisted even after adjusting for smoking and osteoporosis in MVMR.

Numerous observational studies have reported associations between CD or UC and the risk of AIH, yet the potential causality remains complex and uncertain. A recent cohort study, comprising 3,684 patients with UC, reported a prevalence of AIH at 0.24% [17]. Furthermore, the prevalence of AIH was observed to be higher in patients with IBD, particularly manifesting in patients with more severe colitis [38]. AIH occurrences were reported in UC or CD populations, with a higher prevalence commonly associated with UC compared to CD. Our findings have revealed a causal relationship between IBD and AIH. This association appeared to have clinical significance. Evidence suggests that patients with AIH and IBD developed liver disease at a younger age, had a lower remission rate, experienced a higher rate of treatment failure, and were more predisposed to developing cirrhosis [39]. Distinguishing between AIH and PSC during the progression of IBD can be challenging owing to the considerable overlap in clinical features [40]. The association between PSC and AIH is notably prevalent, accounting for approximately 63% of PSC cases in pediatric patients with IBD [41]. Recent MR studies have also supported a positive causal relationship between IBD and both PSC and primary biliary cholangitis, which aligns with our findings [42, 43]. Some authors have suggested that patients with IBD associated with AIH had a more refractory disease course and a higher likelihood of requiring rectal resection [44–46].

Diagnosing AIH does not rely on a single diagnostic test; instead, it involves a comprehensive assessment of clinical, biochemical, serological, immunological, and histological features. The International AIH Group (IAIHG) has established two diagnostic scoring systems for patients with AIH [47, 48]. However, accurately distinguishing AIH from IBD can be challenging owing to the considerable overlap in clinical and pathological features between the two conditions. It has been estimated that approximately half of patients with IBD may exhibit abnormal liver function tests during their illness. These abnormalities can vary from asymptomatic elevation of liver enzymes to the development of chronic liver disease. The underlying causes of abnormal liver function tests in these patients are diverse, including liver disease itself, side effects of treatment, and idiopathic factors. In cases where a definitive diagnosis is required, a liver biopsy is often necessary. However, repeated liver biopsies are often deemed invasive, time-consuming, and burdensome for the patients. Therefore, clinical remission is assessed based on specific biochemical indicators, such as normalized serum transaminase levels and autoantibody titers [49]. Previous research has indicated that testing for anti-neutrophil cytoplasm antibodies (ANCA) may be considered appropriate for suspected patients with AIH-1 who lack traditional autoantibodies, or to differentiate between UC and CD in case of diagnostic uncertainty [50]. Patients with AIH commonly develop atypical peripheral ANCA (pANCA), which specifically targets antigens located at the periphery of the nucleus. Importantly, the revised original scoring system for AIH includes pANCA positivity as an additional parameter for assessments [51–53]. It was reported that the atypical pANCA was frequently detected in patients with AIH-1 with the prevalence ranging from 65% to 81%, whereas atypical pANCA was usually negative in patients with AIH-2 [54]. Furthermore, atypical pANCA can also be detected in 40–70% of patients with UC, although their occurrence is less common in patients with CD. In summary, while the detection of pANCA may complement autoimmune serological markers, it should not replace clinical and imaging-based diagnostic assessments [50, 52].

IBD and intestinal inflammation were closely linked to the risk of AIH. The liver, being a sterile organ, is commonly affected by intestinal inflammation and bacterial translocation. This association is attributed to the disruption of the protective colonic barrier, which facilitates immune activation via the gut-liver axis. A proposed mechanism for the development of AIH in patients with IBD was the hypothesis of a “leaky gut”. This theory suggested that heightened intestinal permeability during intestinal inflammation enables the translocation of bacteria and toxins into the hepatic portal circulation via compromised intestinal mucosa characteristic of IBD. Subsequently, inflammatory cells were activated, leading to the secretion of pro-inflammatory cytokines, thereby triggering immune disorders and liver cell damage [55]. Therefore, the leaky gut hypothesis suggested that liver inflammation in AIH–IBD was linked to microbial composition. Recent studies indicated that patients with AIH had lower bacterial diversity and alterations in gut microbiota composition. Specifically, the reduction of Bifidobacterium in AIH was associated with the failure to attain remission [56, 57]. Importantly, microbiota remodeling can significantly influence the outcome of autoimmune diseases, potentially unveiling novel therapeutic avenues. Probiotics, as adjuvant therapy, could be employed in non-responsive AIH following remission achieved with standard immunosuppression, thereby mitigating relapse and disease progression [58]. Additionally, emerging microbiota-directed therapies offer promising prospects for addressing dysbiosis in AIH [58, 59].

Several studies have suggested that smoking and osteoporosis were shared risk factors for both IBD and AIH [35–37]. To adjust potential confounding from these shared risk factors, MVMR was employed. Subsequent MVMR analysis revealed that the positive direct effect of IBD on the risk of AIH remained statistically significant compared with univariable MR after accounting for smoking and osteoporosis. These results suggested that the observed positive direct effect was independent of the shared risk factors of IBD and the risk of AIH. Such findings reinforce the evidence indicating a lack of association between these shared risk factors and IBD, as reported in previous observational and MR studies. Additionally, several studies have even suggested a protective effect of smoking against UC [60–62]. In terms of mechanisms, it has been reported that the combustion byproducts of tobacco contain dioxide, which can elicit anti-inflammatory and protective responses in UC [63]. Additionally, several authors have suggested that the receptor from immune cells stimulated by nicotine may decrease the production of pro-inflammatory cytokines and suppress the function of regulatory T cells [64].

This MR study elucidated several strengths. Firstly, interference of reverse causality and residual confounders was reduced through MR analysis. Secondly, the study subjects all belonged to European ancestry, thereby minimizing potential bias arising from ethnic heterogeneity. It is pertinent to note some limitations in this study. Firstly, MR results in other populations should be further investigated, although the European ancestry limited the possibility of demographic stratification bias. Secondly, we relaxed the *P*-value threshold between instruments and exposures to obtain more SNPs, potentially violating the first assumption of MR analysis. However, the F statistic for each SNP exceeded 10, indicating an absence of weak instrument bias. Lastly, while MR analysis can infer potential causality, it cannot definitively establish causal relationships. Moreover, there may be other potential shared risk factors apart from cigarettes per day and osteoporosis. Therefore, further research is needed to investigate the role of additional confounders in future studies. This study, despite its limitations, serves as an exploratory hypothesis-generating endeavor, indicating directions for further research. In conclusion, our findings suggest that IBD serves as an important risk factor for the development of AIH.

## Supporting information

S1 Fig (DOCX)

S1 Table (XLS)
