# Supplementary material for: Inflammatory bowel disease and risk of autoimmune hepatitis: A univariable and multivariable Mendelian randomization study
Source: PLoS One. 2024 Jun 7;19(6):e0305220. doi: 10.1371/journal.pone.0305220 (PMC11161122; doi:10.1371/journal.pone.0305220)
Supplement: S1 Fig — (DOCX) [file pone.0305220.s001.docx]

**Inflammatory bowel disease and risk of autoimmune hepatitis: A univariable and multivariable Mendelian randomization study**

# **Supplementary results for primary results**

**1.1 The relationship between crohn's disease and autoimmune hepatitis.**


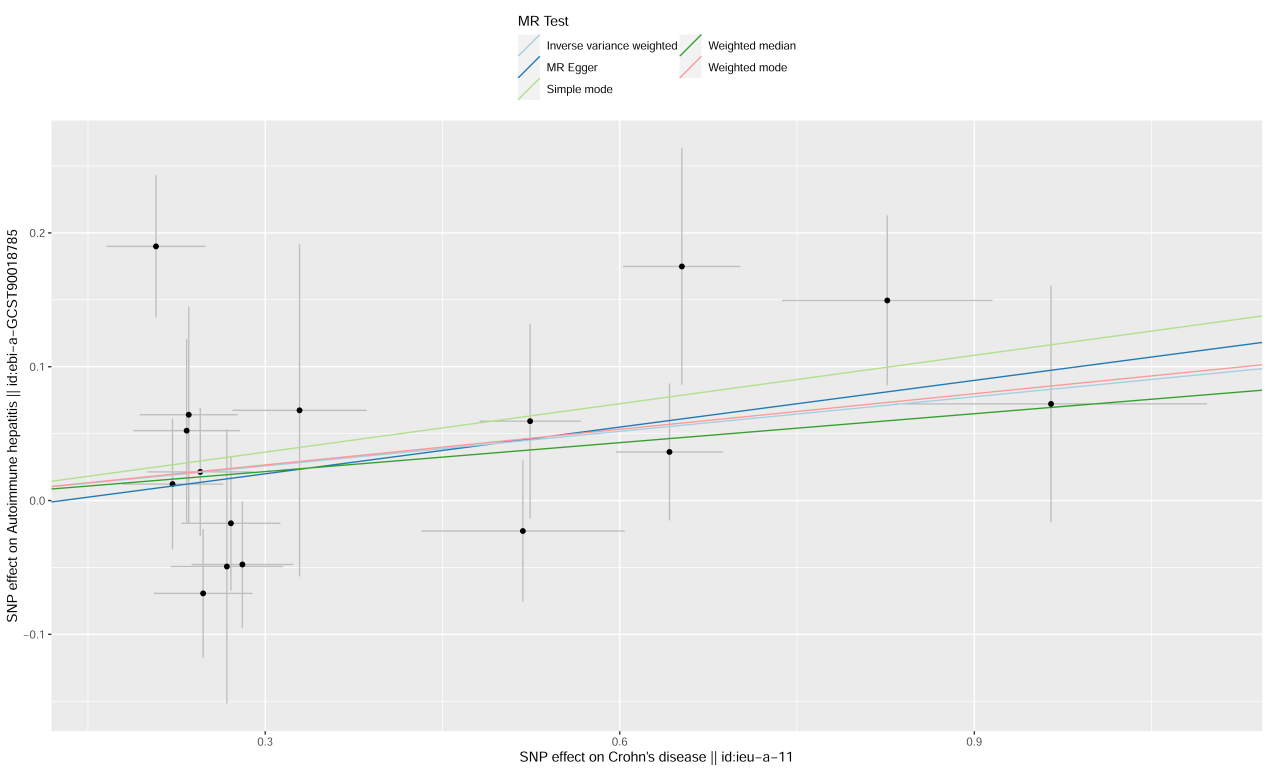


**Supplementary Figure 1.** Scatter plots presenting the relationship between crohn's disease and autoimmune hepatitis in primary result.


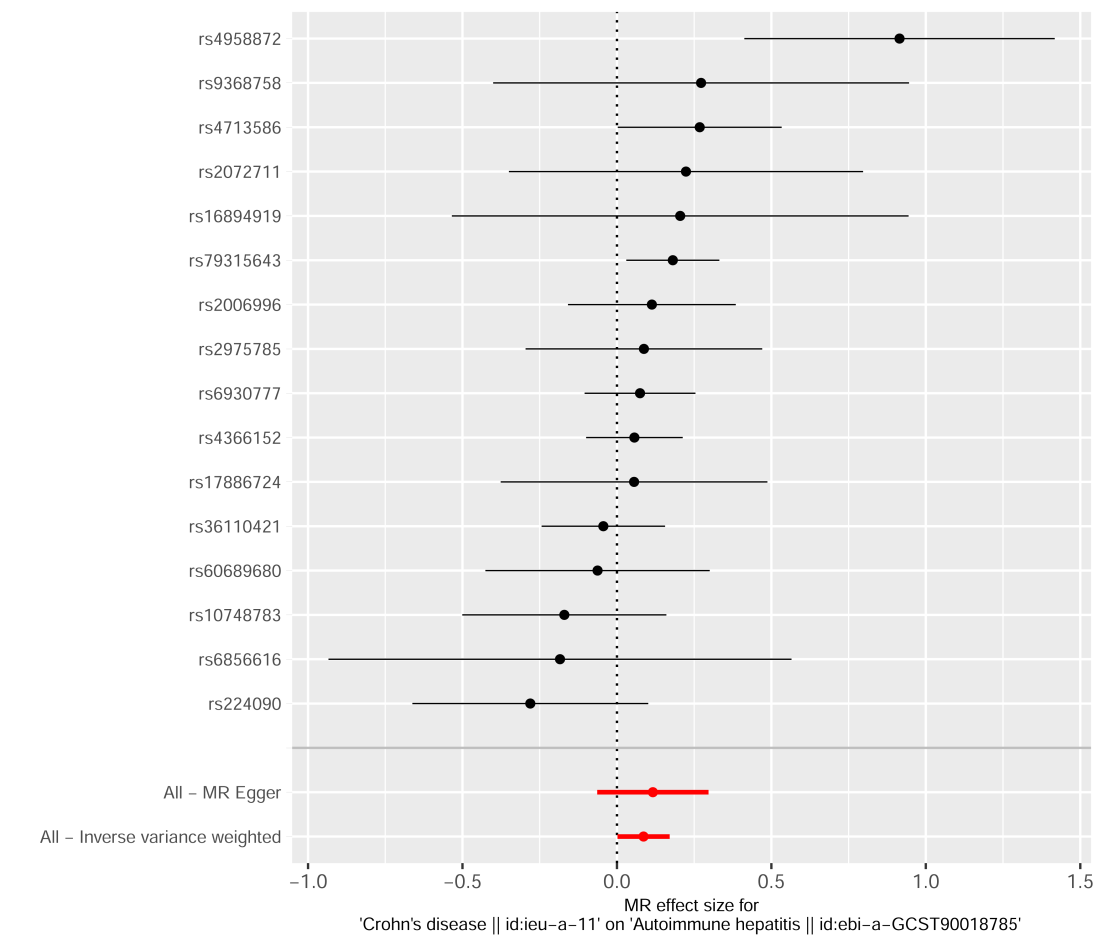


**Supplementary Figure 2.** Forest plots presenting the relationship between crohn's disease and autoimmune hepatitis in primary result.


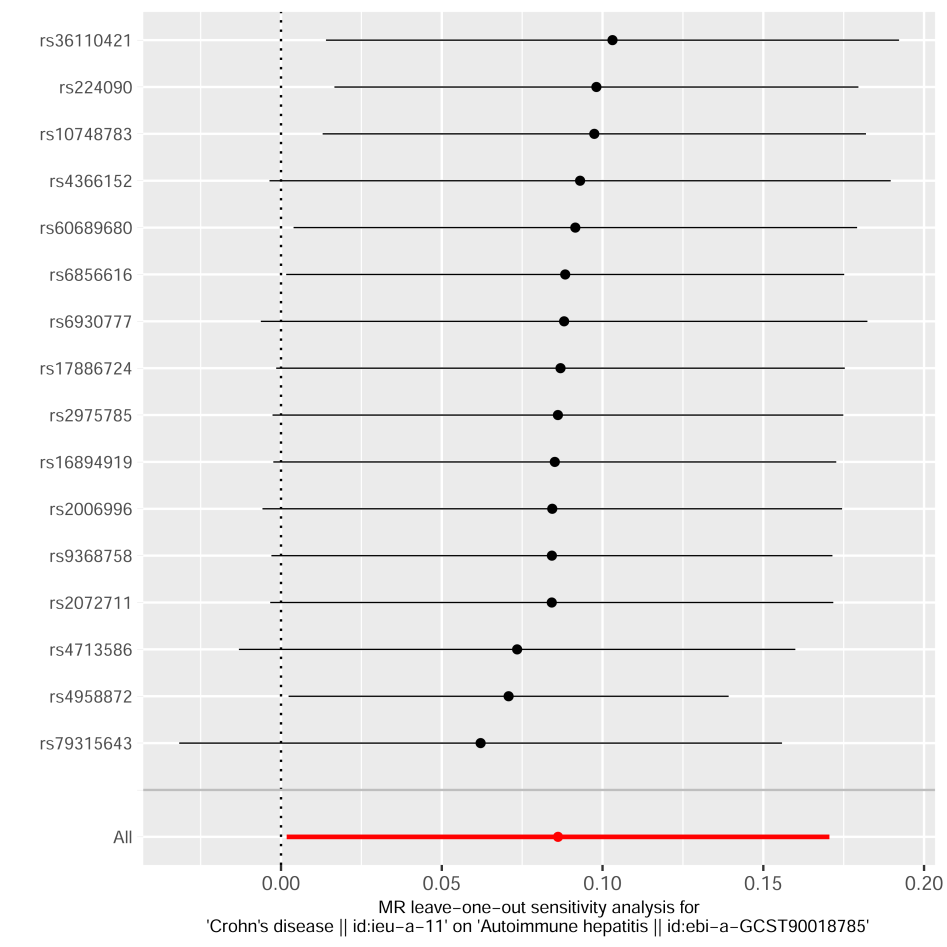


**Supplementary Figure 3.** Leave-one-out plot presenting the relationship between crohn's disease and autoimmune hepatitis in primary result.


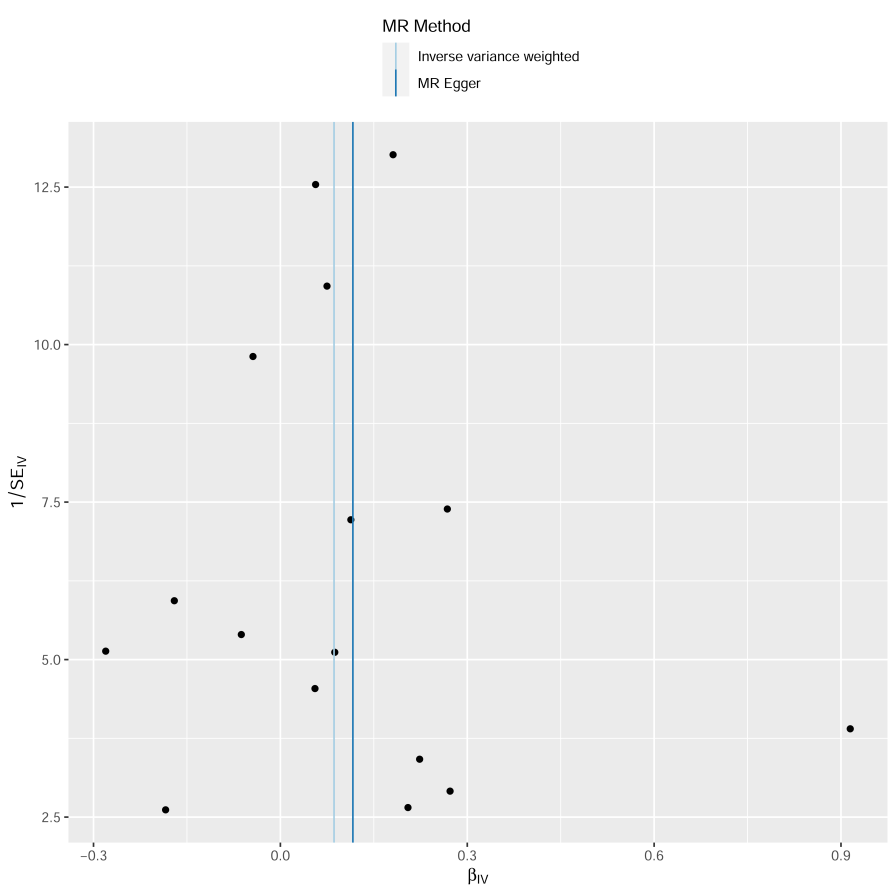


**Supplementary Figure 4.** Funnel plots presenting the relationship between crohn's disease and autoimmune hepatitis in primary result.

**1.2 The relationship between ulcerative colitis and autoimmune hepatitis**


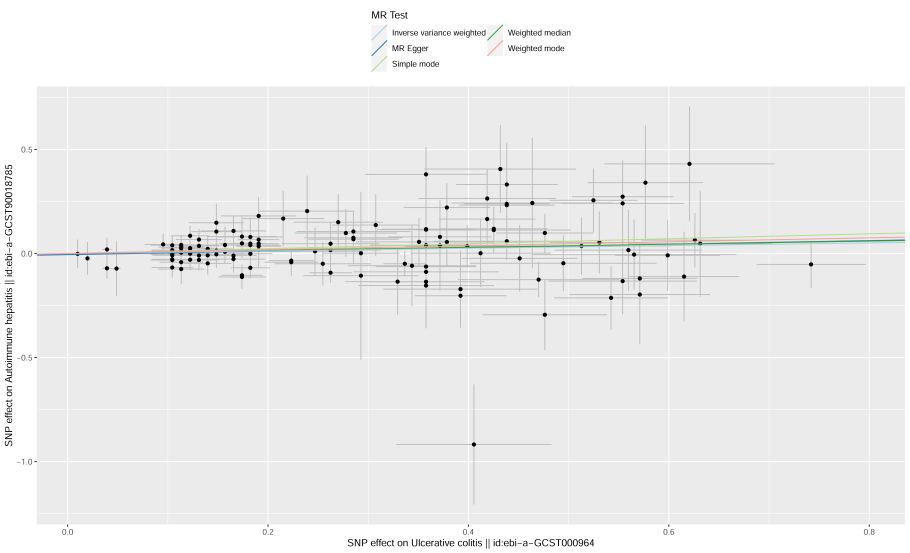


**Supplementary Figure 5.** Scatter plot presenting the relationship between ulcerative colitis and autoimmune hepatitis in primary result.


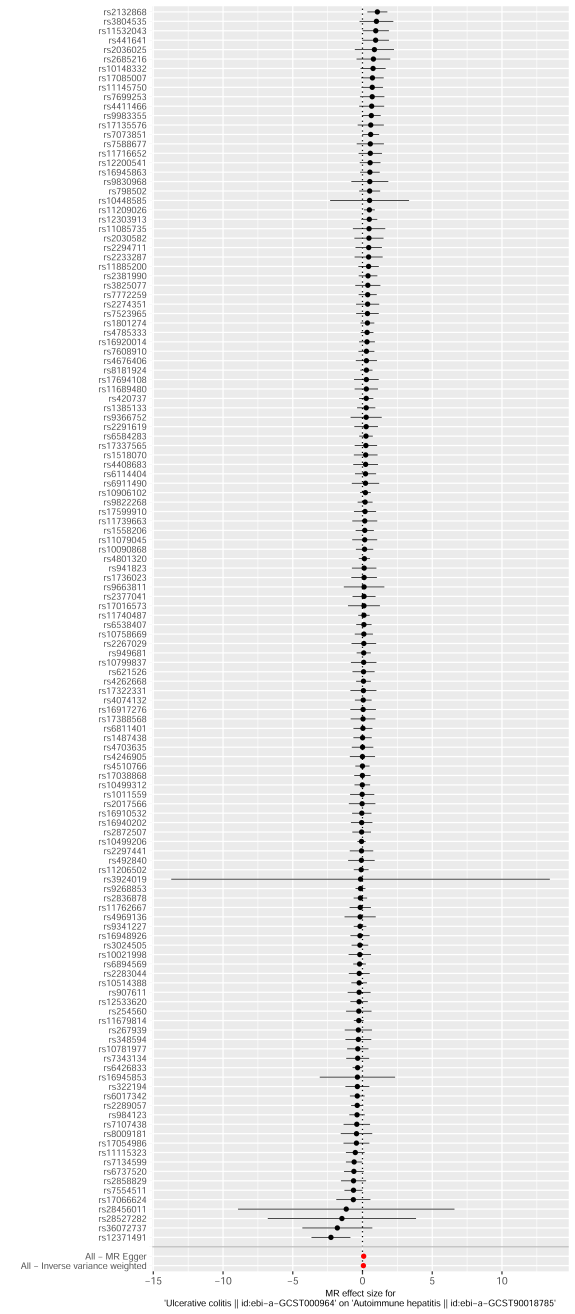


**Supplementary Figure 6.** Forest plot presenting the relationship between ulcerative colitis and autoimmune hepatitis in primary result.


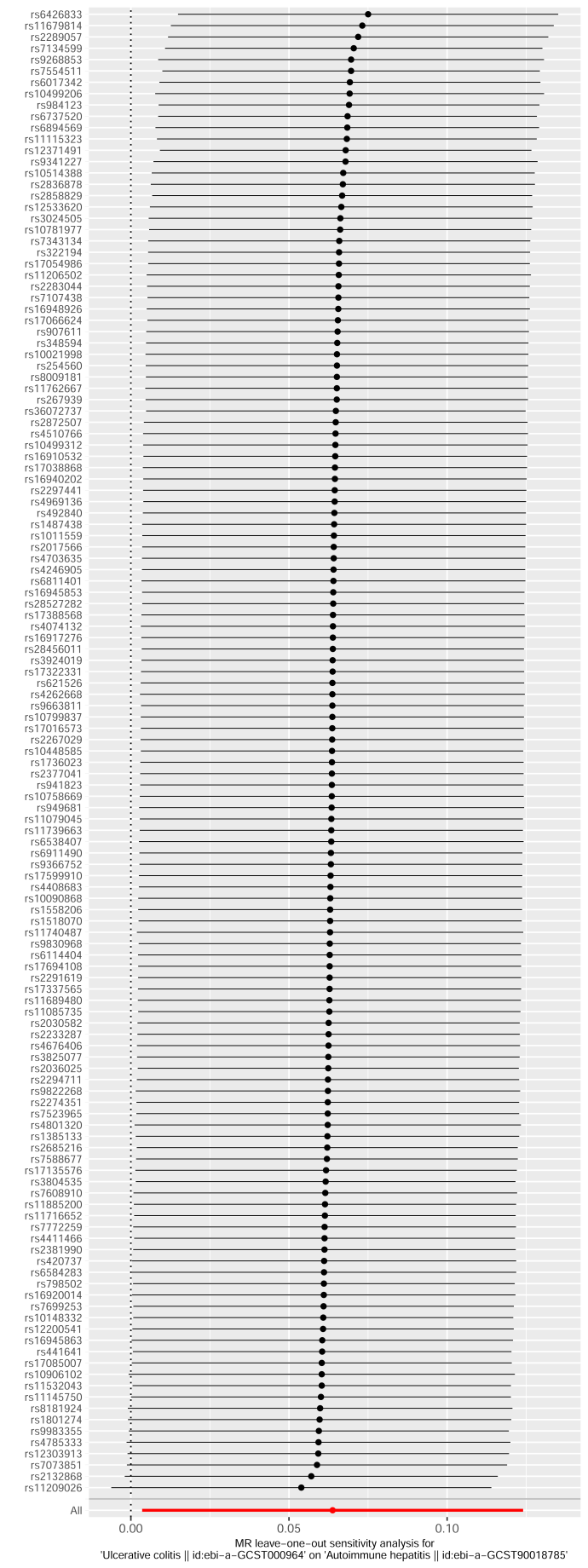


**Supplementary Figure 7.** Leave-one-out plot presenting the relationship between ulcerative colitis and autoimmune hepatitis in primary result.


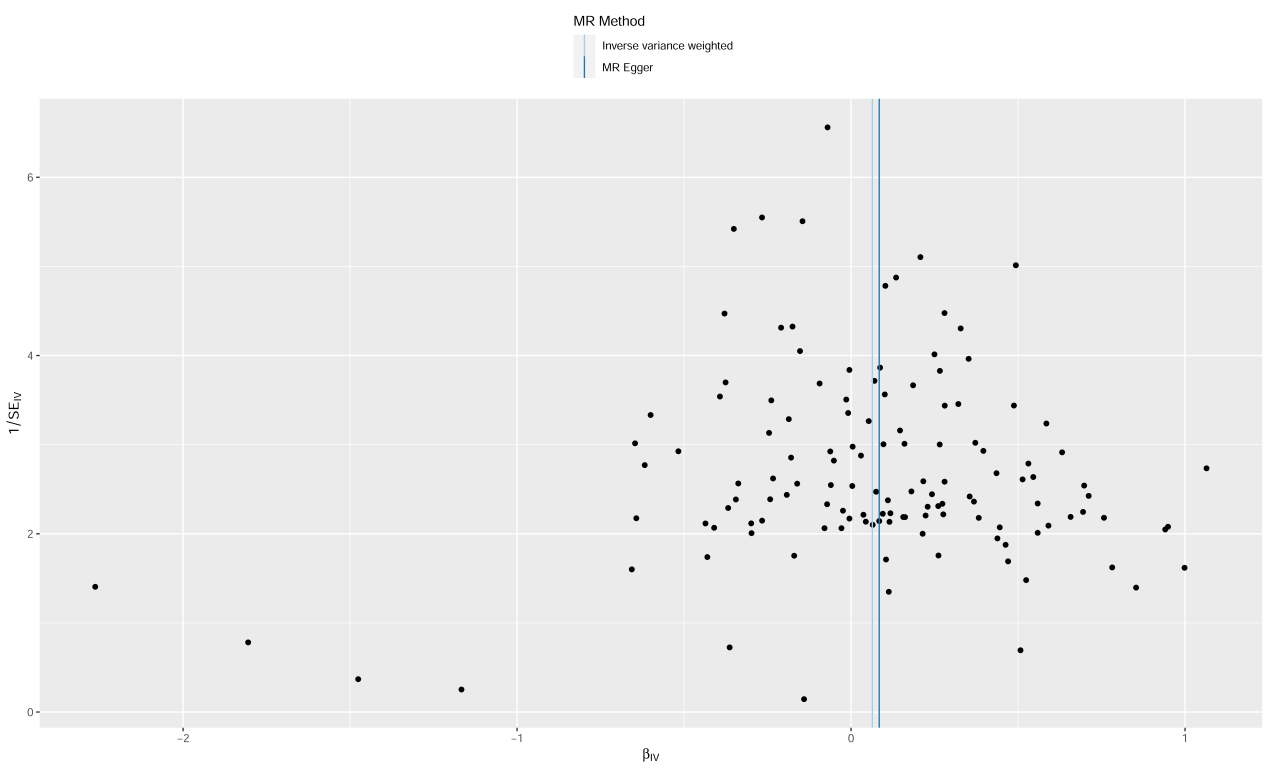


**Supplementary Figure 8.** Funnel plot presenting the relationship between ulcerative colitis and autoimmune hepatitis in primary result.

**1.3 The relationship between inflammatory bowel disease (finn-b-K11_IBD) and autoimmune hepatitis.**


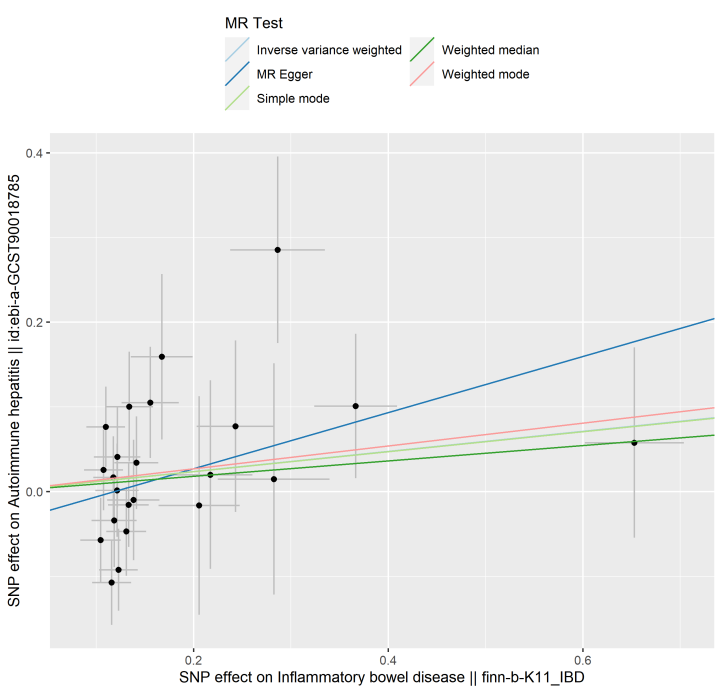


**Supplementary Figure 9.** Scatter plots presenting the relationship between inflammatory bowel disease and autoimmune hepatitis in replication analysis.


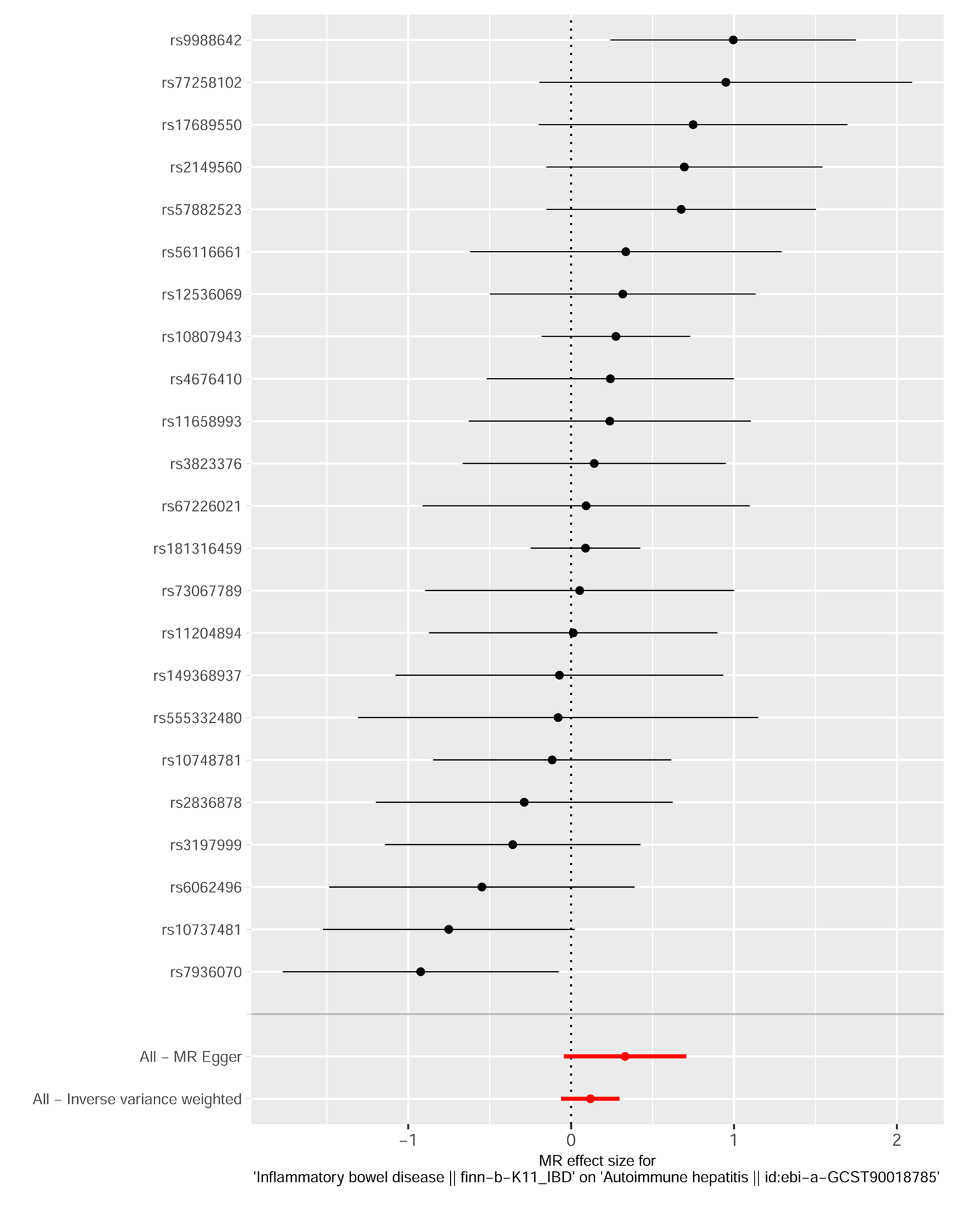


**Supplementary Figure 10.** Forest plots presenting the relationship between inflammatory bowel disease and autoimmune hepatitis in replication analysis.


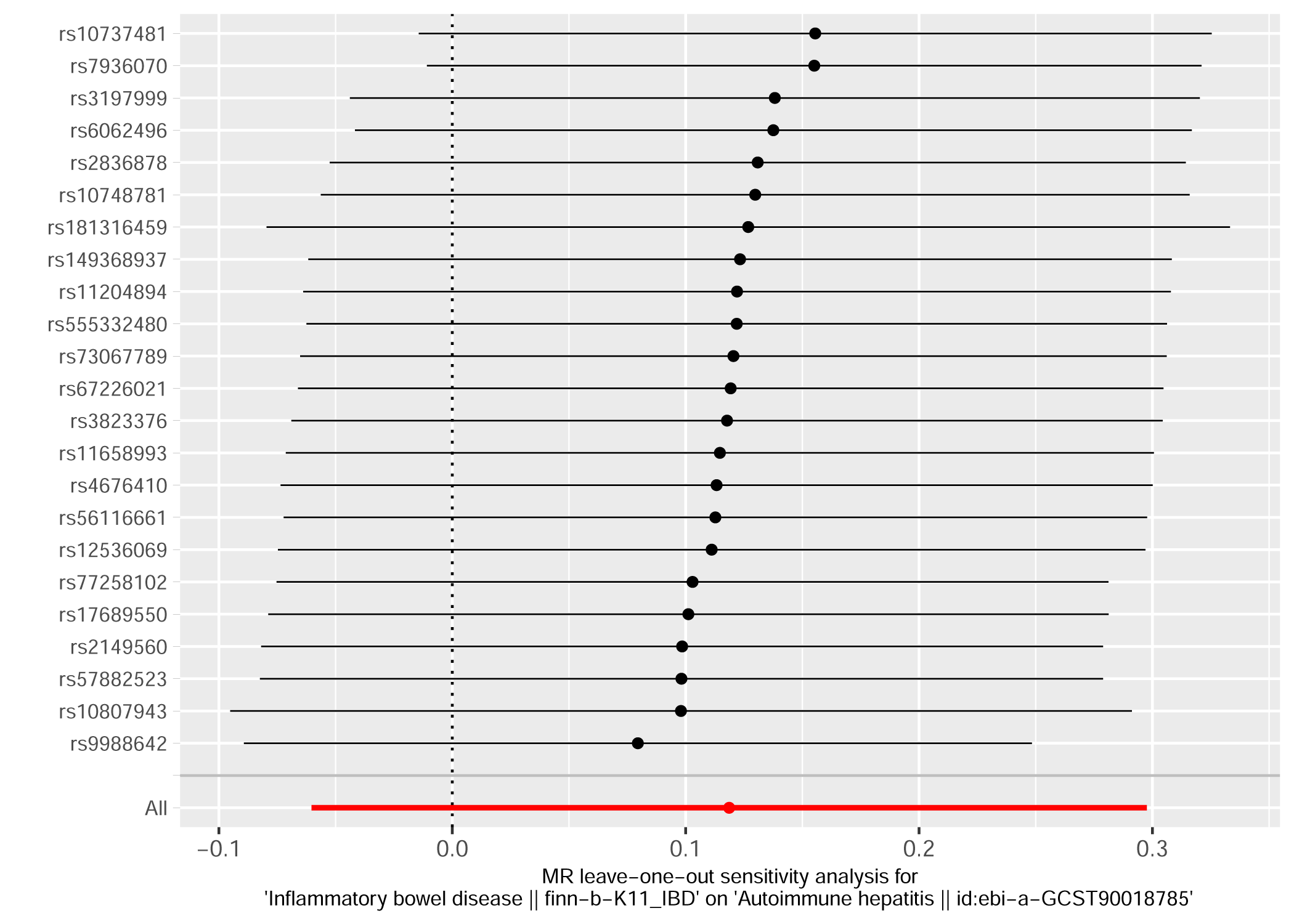


**Supplementary Figure 11.** Leave-one-out plot presenting the relationship between inflammatory bowel disease and autoimmune hepatitis in replication analysis.


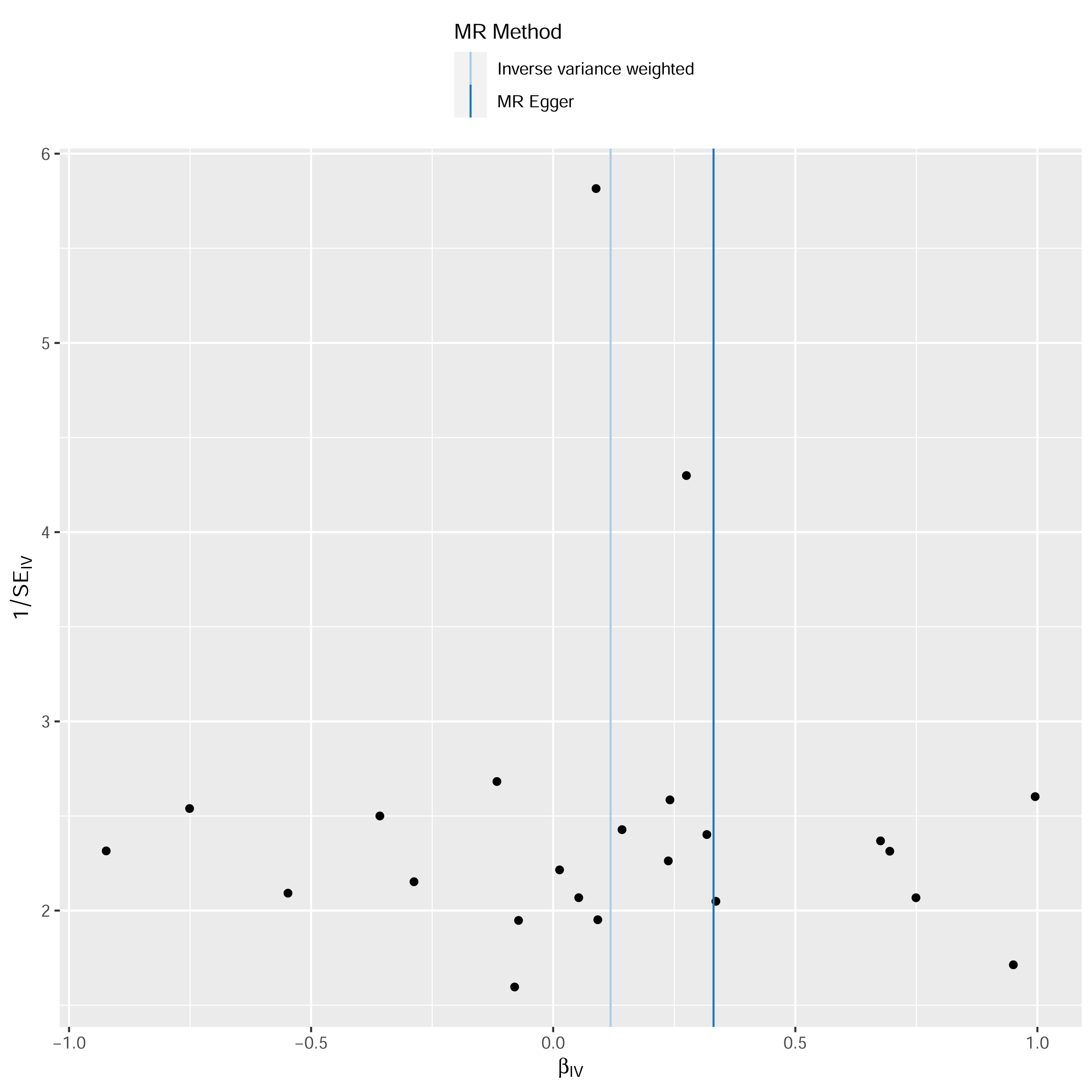


**Supplementary Figure 12.** Funnel plot presenting the relationship between inflammatory bowel disease and autoimmune hepatitis in replication analysis.

# **Supplementary results of replication analysis**

**2.1 The relationship between crohn's disease and autoimmune hepatitis.**


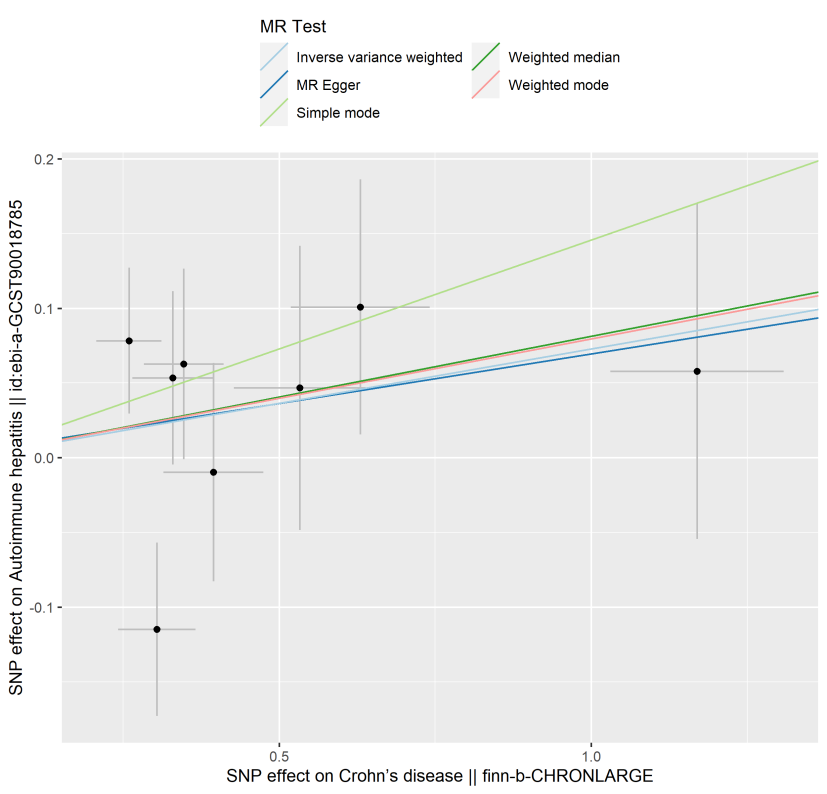


**Supplementary Figure 13.** Scatter plots presenting the relationship between crohn's disease and autoimmune hepatitis in replication analysis.


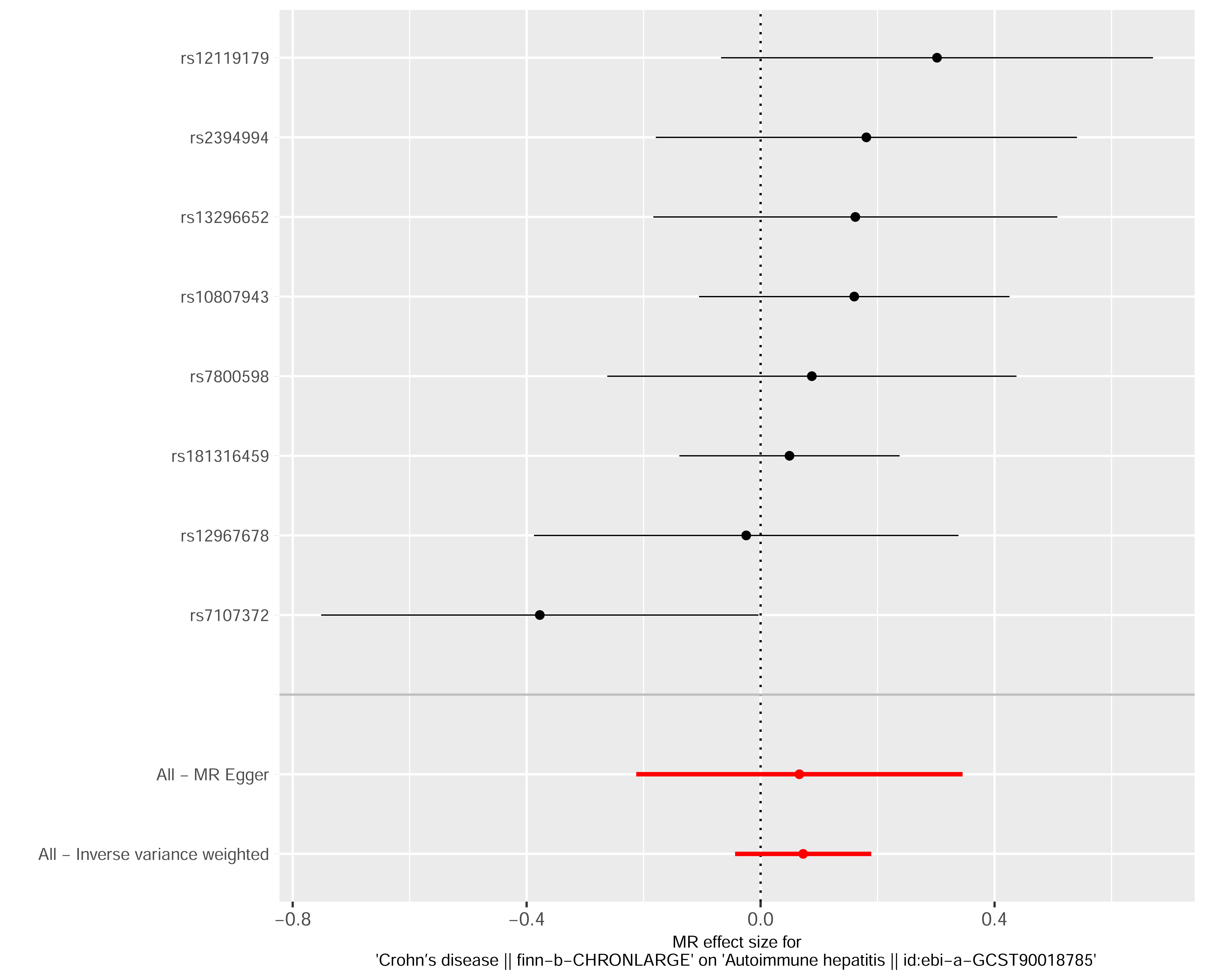


**Supplementary Figure 14.** Forest plots presenting the relationship between crohn's disease and autoimmune hepatitis in replication analysis.


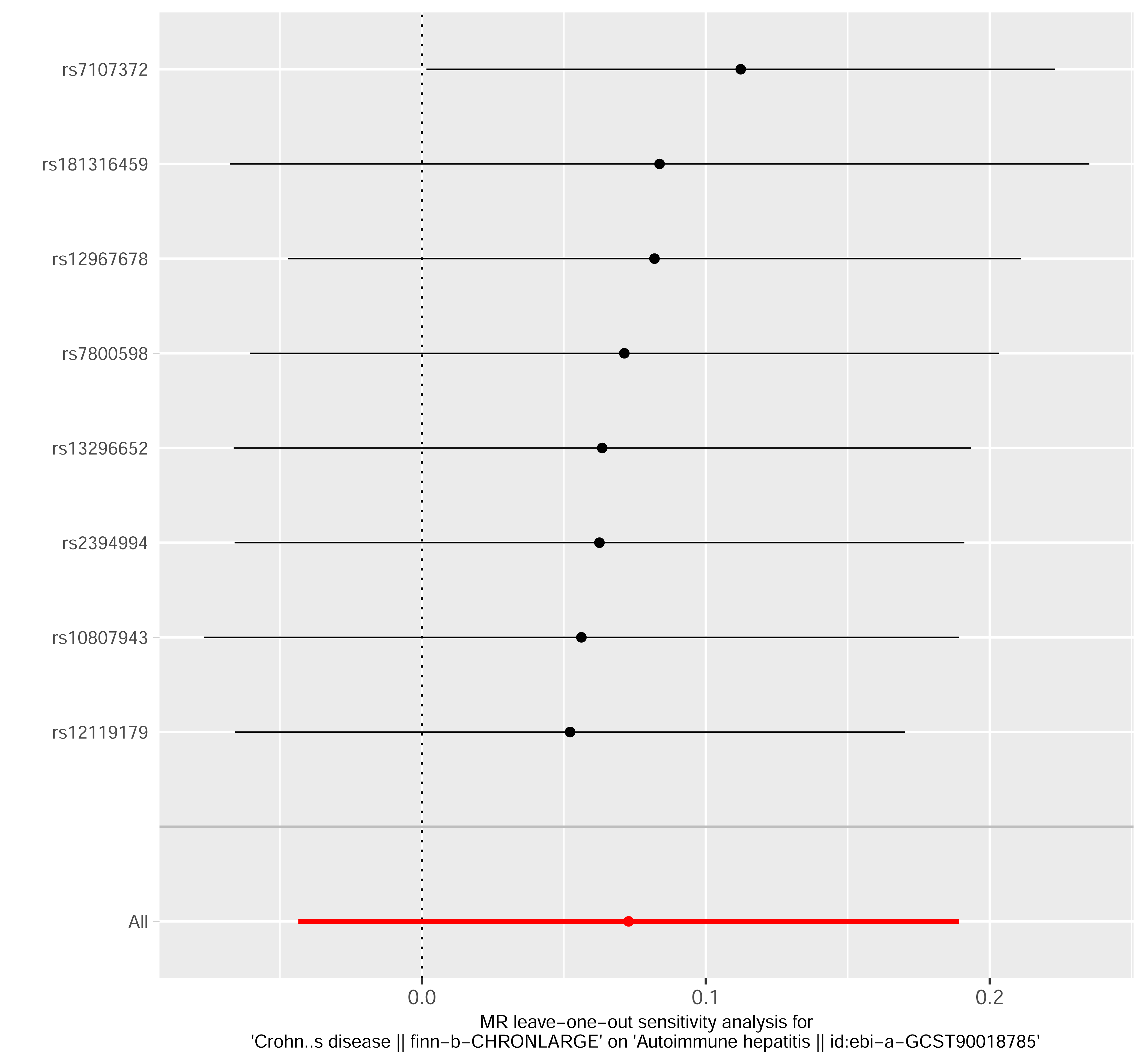


**Supplementary Figure 15.** Leave-one-out plot presenting the relationship between crohn's disease and autoimmune hepatitis in replication analysis.


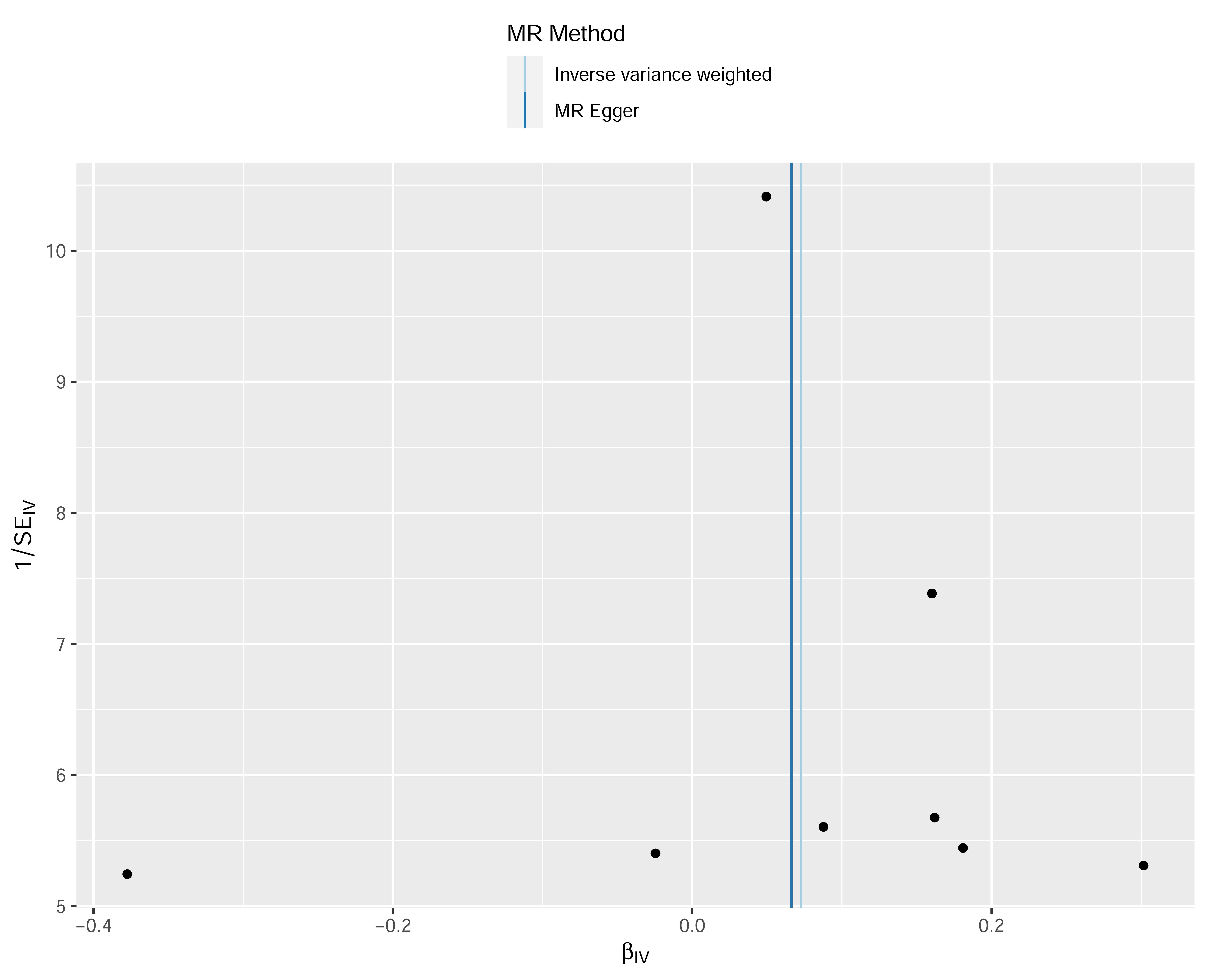


**Supplementary Figure 16.** Funnel plot presenting the relationship between crohn's disease and autoimmune hepatitis in replication analysis.

**2.2 The relationship between ulcerative colitis and autoimmune hepatitis.**


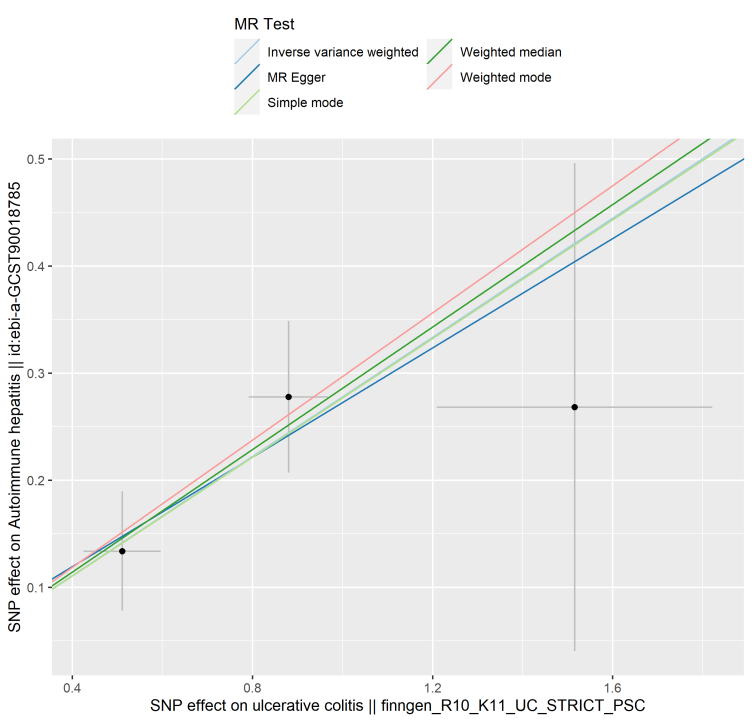


**Supplementary Figure 17.** Scatter plots presenting the relationship between ulcerative colitis and autoimmune hepatitis in replication analysis.


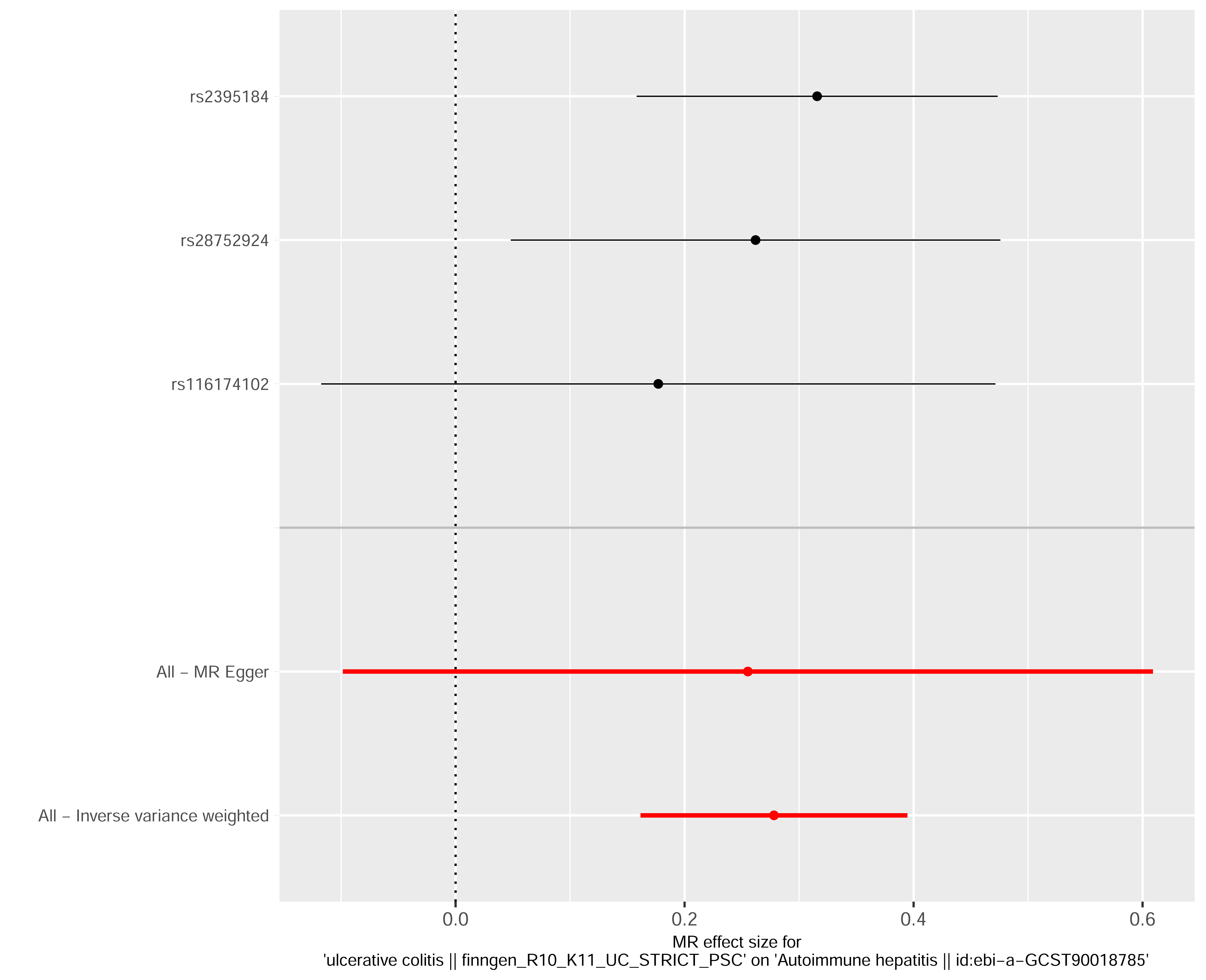


**Supplementary Figure 18.** Forest plots presenting the relationship between ulcerative colitis and autoimmune hepatitis in replication analysis.


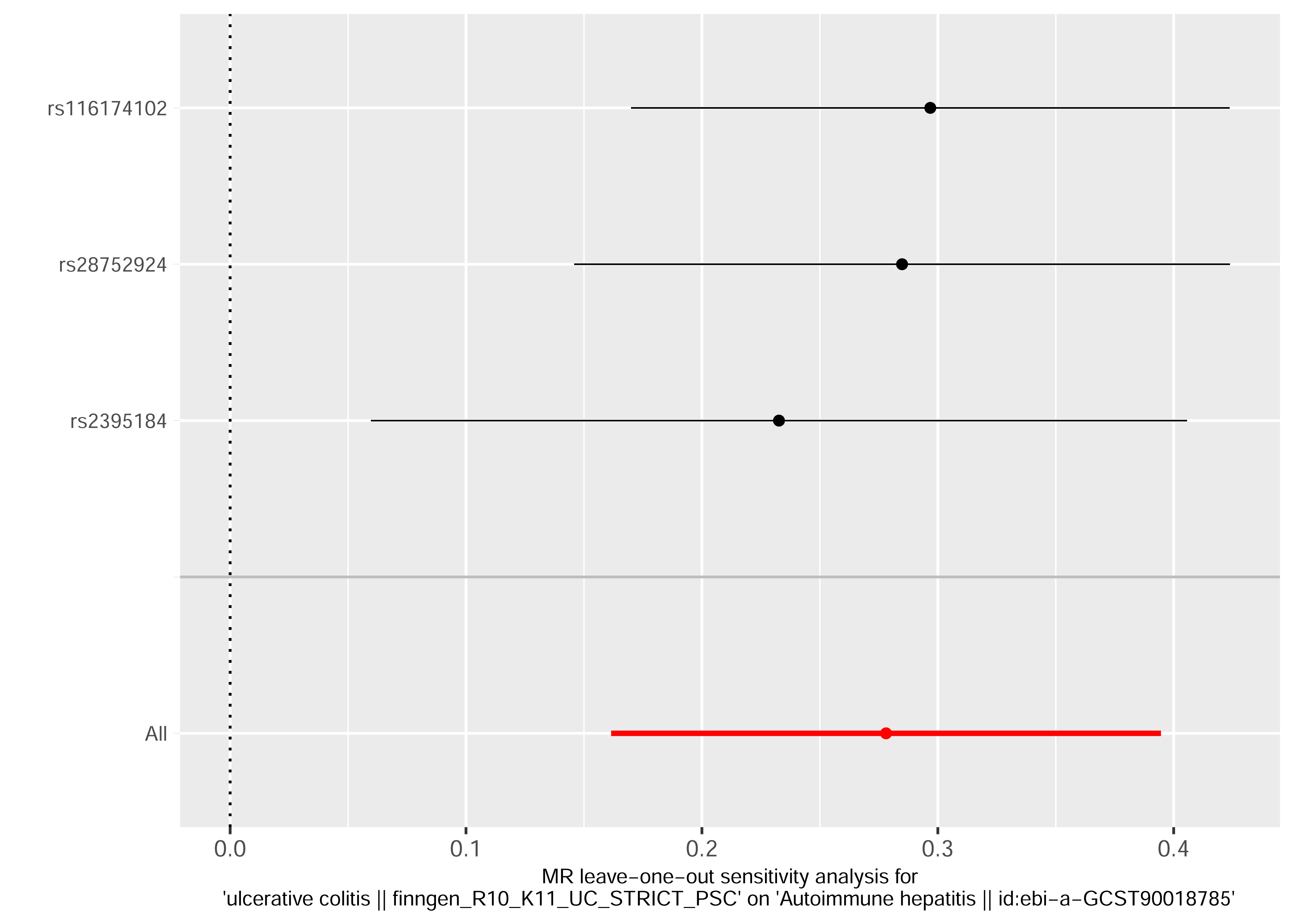


**Supplementary Figure 19.** Leave-one-out plot presenting the relationship between ulcerative colitis and autoimmune hepatitis in replication analysis.


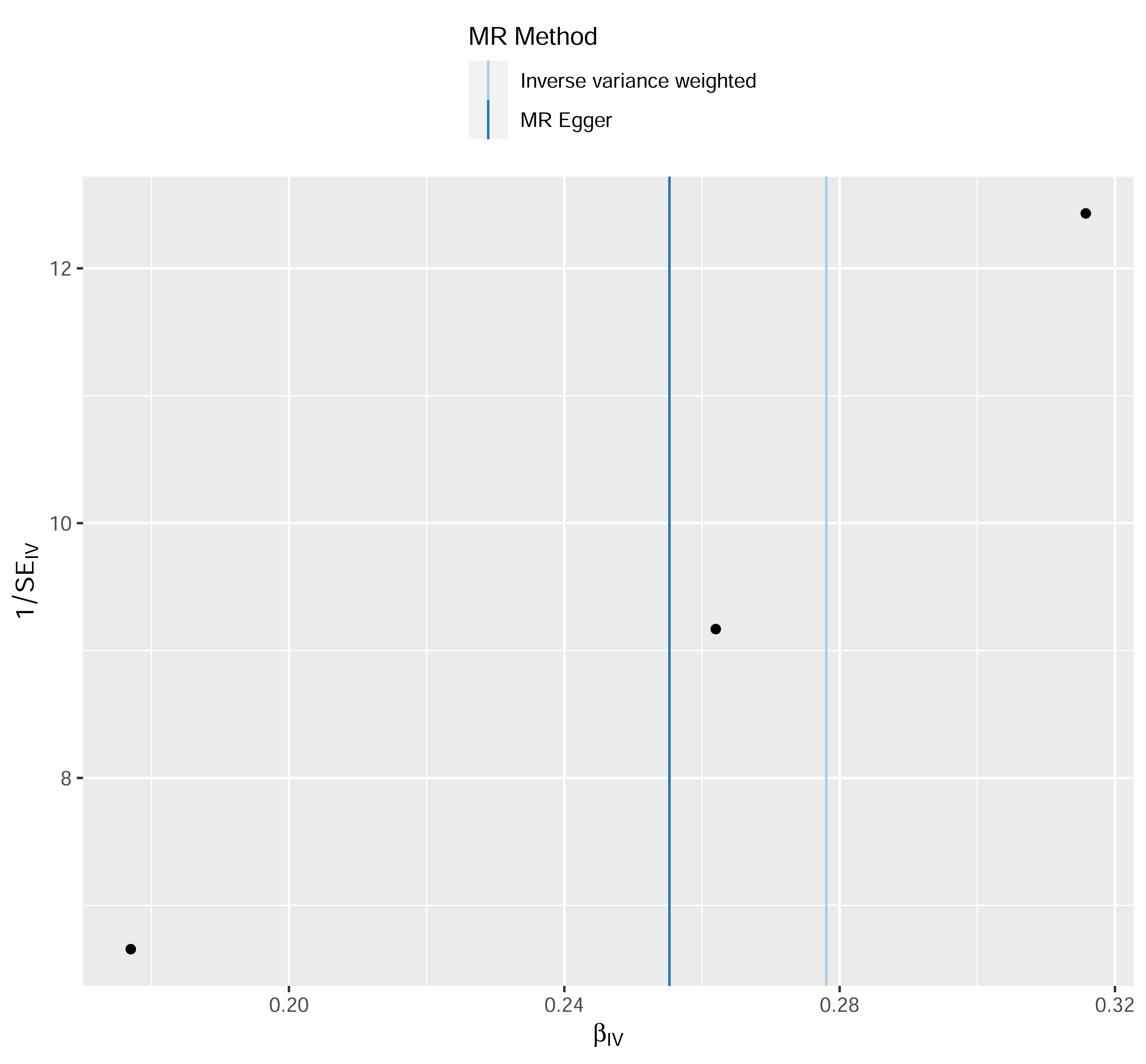


**Supplementary Figure 20.** Funnel plot presenting the relationship between ulcerative colitis and autoimmune hepatitis in replication analysis.

**2.3 The relationship between inflammatory bowel disease (ebi-a-GCST004131) and autoimmune hepatitis.**

**
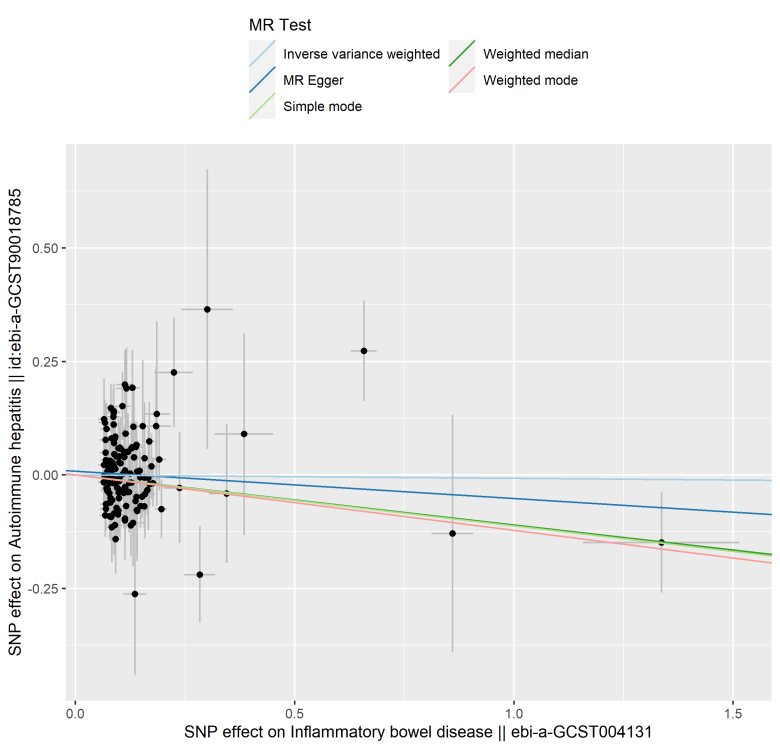
**

**Supplementary Figure 21.** Scatter plots presenting the relationship between inflammatory bowel disease and autoimmune hepatitis in replication analysis.


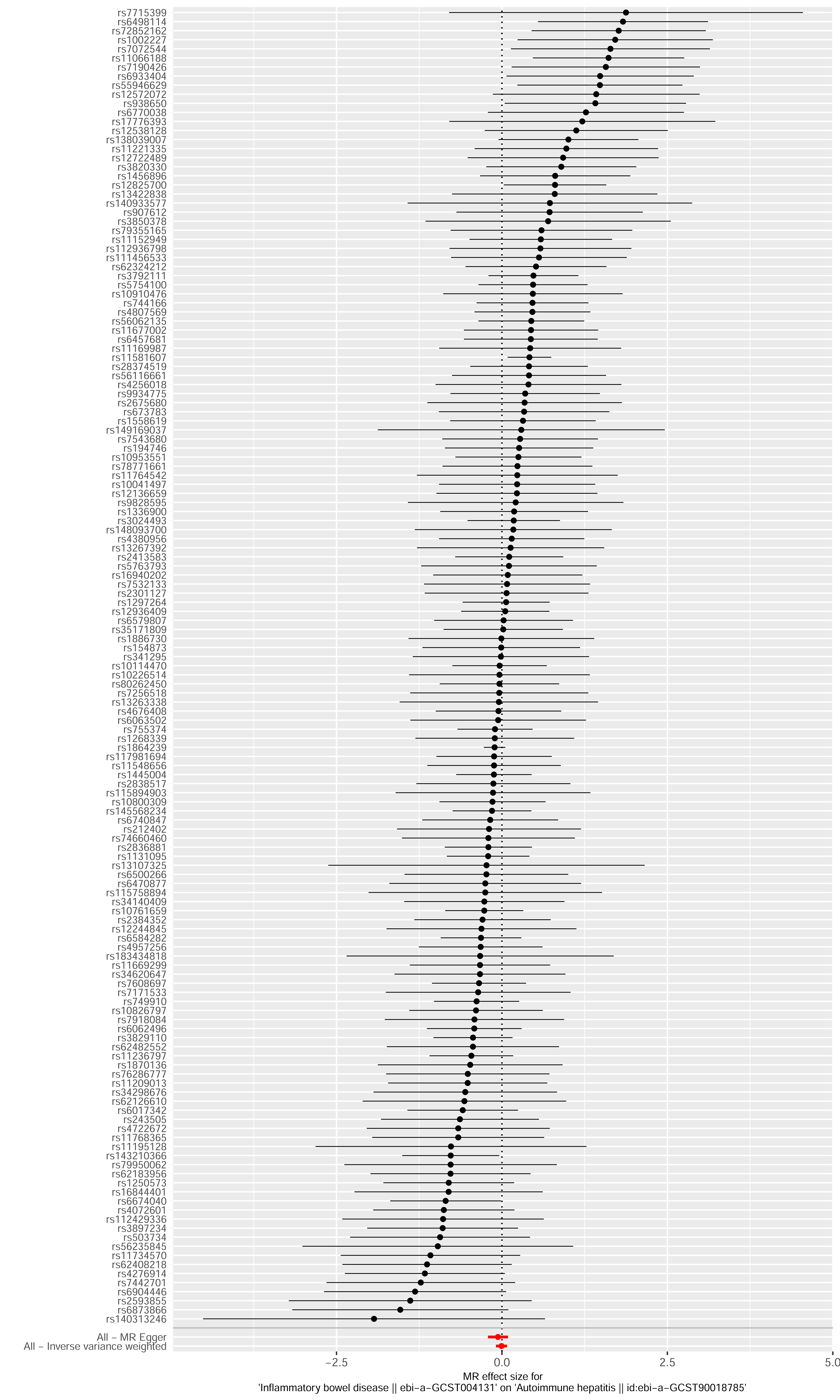


**Supplementary Figure 22.** Forest plots presenting the relationship between inflammatory bowel disease and autoimmune hepatitis in replication analysis.


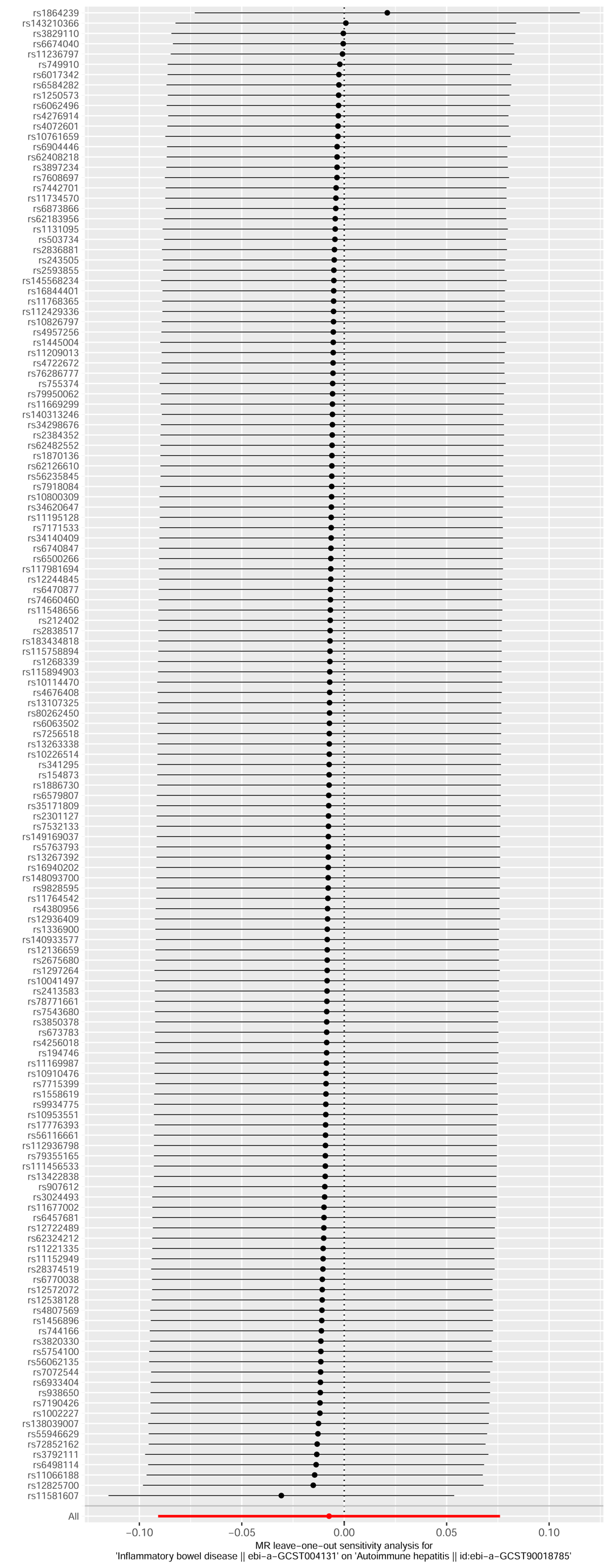


**Supplementary Figure 23.** Leave-one-out plot presenting the relationship between inflammatory bowel disease and autoimmune hepatitis in replication analysis.


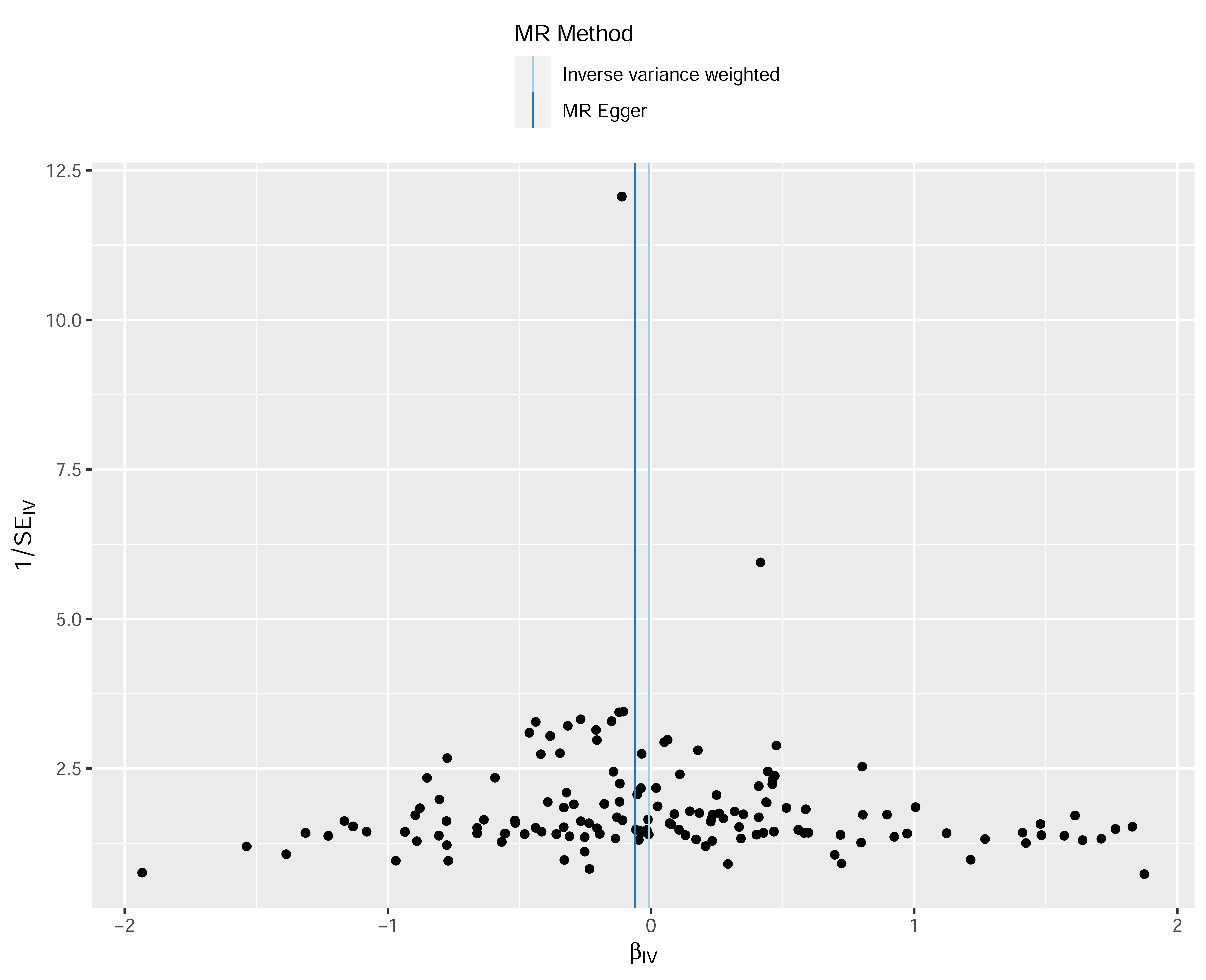


**Supplementary Figure 24.** Funnel plot presenting the relationship between inflammatory bowel disease and autoimmune hepatitis in replication analysis.
